# Predictors of Bleeding Complications After Extracorporeal Cardiopulmonary Resuscitation: Insights From the SAVE-J II Study

**DOI:** 10.1016/j.jacasi.2025.09.027

**Published:** 2025-12-19

**Authors:** Madoka Sano, Toshiaki Toyota, Yoshinori Matsuoka, Hirohiko Kohjitani, Yusuke Watanabe, Yuta Azumi, Hideyuki Hayashi, Ryosuke Murai, Junichi Ooka, Yasuhiro Sasaki, Tomohiko Taniguchi, Kitae Kim, Atsushi Kobori, Natsuhiko Ehara, Makoto Kinoshita, Akihiko Inoue, Toru Hifumi, Tetsuya Sakamoto, Yasuhiro Kuroda, Yosuke Yamamoto, Koichi Ariyoshi, Yasushi Okuno, Koh Ono, Yutaka Furukawa, SAVE-J II Study Group

**Affiliations:** a Department of Cardiovascular Medicine, Kobe City Medical Center General Hospital, Kobe, Japan; b Department of Emergency Medicine, Kobe City Medical Center General Hospital, Kobe, Japan; c Department of Healthcare Epidemiology, Graduate School of Medicine and Public Health, Kyoto University, Kyoto, Japan; d Department of Cardiovascular Medicine, Kyoto University Graduate School of Medicine, Kyoto, Japan; e Department of Artificial Intelligence in Healthcare and Medicine, Kyoto University Graduate School of Medicine, Kyoto, Japan; f Department of Emergency and Critical Care Medicine, Hyogo Emergency Medical Center, Kobe, Japan; g Department of Emergency and Critical Care Medicine, St. Luke’s International Hospital, Tokyo, Japan; h Teikyo University School of Medicine, Department of Emergency Medicine, Tokyo, Japan; i Department of Emergency, Disaster and Critical Care Medicine, Kagawa University Hospital, Kagawa, Japan

**Keywords:** extracorporeal cardiopulmonary resuscitation (ECPR), hemorrhagic complications, out-of-hospital cardiac arrest (OHCA)

## Abstract

**Background:**

Extracorporeal cardiopulmonary resuscitation (ECPR), an emerging resuscitative therapy following refractory cardiac arrests, is associated with hemorrhagic complications that potentially affect patient outcomes.

**Objectives:**

This study evaluated the risks and predictors of hemorrhagic complications among patients who underwent ECPR for out-of-hospital cardiac arrest (OHCA) from different causes.

**Methods:**

Using the SAVE-J II (Study of Advanced Cardiac Life Support for Ventricular Fibrillation with Extracorporeal Circulation in Japan) study, we analyzed multicentric data of patients who underwent ECPR for OHCA from 2013 to 2018 in Japan. Based on the causes of OHCA, the participants were stratified into endogenous cardiac, endogenous noncardiac, and exogenous groups. The primary outcome was any bleeding.

**Results:**

Among 1,935 patients, 1,417, 305, and 213 patients had endogenous cardiac, endogenous noncardiac, and exogenous causes, respectively. For survivors, the median follow-up period was 36 days, and most of the bleeding events occurred within 1 week post-ECPR. The 30-day cumulative incidence of any bleeding significantly differed among the 3 groups (endogenous cardiac: n = 321 [25.9%]; endogenous noncardiac: n = 41 [18.9%]; and exogenous: n = 27 [13.7%]; *P* < 0.001). However, the risks for bleeding complications did not differ between the causes of OHCA after adjustment for confounders. Intra-aortic balloon pumping use was associated with higher risks of bleedings and lower risk for all-cause death.

**Conclusions:**

Underlying causes of OHCA did not significantly impact adjusted bleeding risks. Intra-aortic balloon pumping use was independently associated with higher bleeding risks and lower mortality, although this warrants cautious interpretation because of a potential selection bias. Vigilant monitoring for bleeding complications is crucial in ECPR patients, especially in those with additional circulatory support devices.

With the increasing number of patients experiencing out-of-hospital cardiac arrest (OHCA), extracorporeal cardiopulmonary resuscitation (ECPR) has emerged as a valuable intervention for patients with refractory cardiac arrest.^1^ ECPR with venoarterial extracorporeal membrane oxygenation (VA-ECMO) support in patients with OHCA ensures sufficient organ perfusion and oxygen supply. Therefore, the adaptation of ECPR is expected to improve survival rates or neurological outcomes of patients with OHCA compared with treatment with conventional cardiopulmonary resuscitation (CPR) only.^2^, ^3^, ^4^, ^5^ However, ECPR is a highly invasive intervention that is associated with various complications, including thrombosis, hemolysis, limb ischemia, infection, and bleeding events.^3^

Among them, bleeding is the most common complication and is associated with increased mortality rates.^6^, ^7^, ^8^ The multifactorial causation of bleeding risk during ECPR includes the requirement for anticoagulation, insertion of large cannulas, CPR-related trauma, and coagulopathy associated with postcardiac arrest syndrome. The use of additional mechanical circulatory support devices, such as intra-aortic balloon pump (IABP), could reduce the cardiac afterload and cardiac burden although it could adversely increase the bleeding risk. The underlying causes of OHCA are varied and may be attributed to the patient’s clinical outcomes. Despite the increasing use of ECPR, there remains a critical knowledge gap regarding the influence of different underlying causes of cardiac arrest on bleeding risk. Understanding the factors that predispose patients to bleeding during ECPR is crucial for optimizing patient management and improving outcomes. However, comprehensive data on associated risk factors in ECPR patients remain scarce.

From the SAVE-J II (Study of Advanced Cardiac Life Support for Ventricular Fibrillation with Extracorporeal Circulation in Japan) study database, an analysis was performed to identify the risk factors for bleeding during the first day of admission and to comprehensively describe details of bleeding during hospitalization in patients with cardiogenic OHCA undergoing ECPR.^9^ They reported that 22.1% experienced bleeding predominantly at the cannulation site and that platelet count of below 100,000/μL on admission was an independent risk factor for early bleeding events. As shown in this study, we frequently experience cannulation-site severe bleeding in patients using large bore devices like ECMO soon after admission. On the other hand, we also experience severe bleeding after the first day of admission and nonprocedure-related bleeding. We reported that additional mechanical circulatory device use would increase bleeding events in patients with cardiogenic shock treated by percutaneous ventricular assist device.^10^ To assess longer-term bleeding events and the effect of additional cardiac support devices on bleeding, including patients with noncardiogenic OHCA, we performed a current analysis.

We hypothesized that bleeding risk in patients with ECPR would differ among the underlying causes of OHCA or the use of mechanical circulatory support devices. In this study, we aimed to evaluate the incidence and predictors of bleeding complications in patients undergoing ECPR, with specific attention to OHCA etiology and concurrent use of mechanical circulatory support. Through a better understanding of these relationships, we sought to identify opportunities for improving risk stratification and patient outcomes.

## Methods

### Study design

The SAVE-J II study is a multicenter retrospective registry conducted in Japan, with 36 participating institutions,^11^ and includes patients aged 18 years or older who were admitted to the emergency department with OHCA and received ECPR between January 2013 and December 2018. The exclusion criteria for the current analysis were as follows: patients who received VA-ECMO after intensive care unit admission; those who were withdrawn after cannulation because of the return of spontaneous circulation; those who achieved return of spontaneous circulation at hospital arrival and ECMO initiation; those who were transferred from other hospitals; and patients who had unknown outcomes, including bleeding complications.

The patients were stratified into 3 groups based on the underlying causes of OHCA as follows: endogenous cardiac, endogenous noncardiac, and exogenous. The primary outcome was any bleeding. The secondary outcomes included bleeding related to the procedure, at cannulation sites, and nonprocedure-related bleeding. Any bleeding was defined as cases requiring blood transfusion, interventional radiology, or surgical hemostasis. Bleeding events were classified into 2 prespecified categories: procedure- and nonprocedure-related bleeding. In detail, procedure-related bleeding included bleeding at cannulation sites, retroperitoneum, and puncture sites excluding ECMO cannulation; and nonprocedure-related bleeding was comprised of bleeding at the brain, upper airway, chest (including mediastinal bleeding, hemothorax, pulmonary hemorrhage, and so on), abdomen (including gastrointestinal tract bleeding, liver, spleen, and abdominal cavity), and other sites. Blood transfusion was defined as the administration of packed red blood cells beyond what was required for routine ECMO circuit maintenance, such as consumption through ECMO device.

The current analysis was performed as a part of the data from SAVE-J II registry, which was registered at the University Hospital Medical Information Network Clinical Trials Registry and the Japanese Clinical Trial Registry (UMIN000036490), and was approved by the Institutional Review Board of Kagawa University (approval number: 2018–110) and each participating institution, including Kobe City Medical Center General Hospital (approval number: zn200304). Informed consent from the patients was waived because of the retrospective nature of the study design, and all procedures were performed in accordance with the ethical standards of the review board of Kobe City Medical Center General Hospital on human experimentation and with the Declaration of Helsinki of 1975.

### Statistical analysis

Continuous variables were expressed as median (IQR) or mean ± SD and were compared using the Mann-Whitney *U* test or Student's *t*-test based upon their distribution. For comparisons among 3 groups, the Kruskal-Wallis test was used. Categorical variables were expressed as numbers and percentages and compared with the chi-square test or Fisher exact test as appropriate. Participant institutions were classified into 4 groups (institution Q1 to Q4) using quartiles in descending order of their ECPR cases. The cumulative incidences of clinical outcomes were estimated using the Kaplan-Meier method, and differences among each group were assessed using a log-rank test. The multivariable Cox proportional hazards assumptions were conducted for the primary outcome measure. We selected 11 clinically relevant risk-adjusting variables listed in Table 1, a priori: OHCA causes, IABP use, age, estimated glomerular filtration rate, hemoglobin, platelet, C-reactive protein, lactate, use of antithrombotic agents before admission, catheter laboratory puncture, and participated institution category. These covariates were selected based on clinical relevance and prior literature, not on statistical significance in univariate analysis. We also planned a sensitivity analysis before implementing statistical analyses to confirm the robustness of our findings by adding the following covariates to the model: activated partial thromboplastin time, fibrinogen, albumin, hypertension, diabetes, chronic renal failure, cardiac disease, cerebrovascular disease, and bystander CPR. Proportional hazards assumptions for the risk-adjustment variables, including categorized OHCA causes, were evaluated using plots of log (time) vs log (−log[survival]) stratified by the variables and deemed acceptable.

**Table 1:** Patient Characteristics Stratified by Strata

	All (N = 1,935)	Endogenous Cardiac Stratum (n = 1,417)	Endogenous Noncardiac Stratum (n = 305)	Exogenous Stratum (n = 213)	*P* Value
Age, y	58.9 ± 14.0	58.8 ± 13.4	58.8 ± 14.3	59.5 ± 16.9	0.72
Age ≥75 y^a^	239 (12.4)	154 (10.9)	41 (13.4)	44 (20.7)	<0.001
Male	1,597 (82.5)	1,229 (86.7)	215 (70.5)	153 (71.8)	<0.001
Body mass index, kg/m^2^	24.7 ± 4.4	24.8 ± 4.2	24.8 ± 5.2	23.9 ± 5.0	0.10
Comorbidity					
Hypertension^b^	755 (29.8)	453 (32.0)	78 (25.6)	46 (21.6)	0.002
Diabetes mellitus	367 (19.0)	307 (21.7)	27 (8.9)	33 (15.5)	<0.001
Dyslipidemia	204 (10.5)	180 (12.7)	14 (4.6)	10 (4.7)	<0.001
Cardiovascular disease^b^	443 (22.9)	384 (27.1)	31 (10.2)	28 (13.2)	<0.001
Cerebrovascular disease^b^	123 (6.4)	92 (6.5)	16 (5.3)	15 (7.0)	0.66
Chronic renal failure^b^	93 (4.8)	77 (5.4)	10 (3.3)	6 (2.8)	0.10
Medication					
Antiplatelet	212 (11.0)	184 (13.0)	16 (5.3)	12 (5.6)	<0.001
Anticoagulant	101 (5.2)	80 (5.7)	10 (3.3)	11 (5.2)	0.24
Antiplatelet or anticoagulant^a^	245 (12.7)	208 (14.7)	20 (6.6)	17 (8.0)	<0.001
Antiplatelet and anticoagulant	34 (1.8)	28 (2.0)	3 (1.0)	3 (1.4)	0.45
No antiplatelet and anticoagulant	1,656 (85.6)	1,181 (83.4)	282 (92.5)	193 (85.6)	<0.001
Location of cardiac arrest					<0.001
Home	785 (40.7)	551 (39.0)	143 (47.0)	91 (42.9)	
Workplace	206 (10.7)	163 (11.5)	27 (8.9)	16 (7.6)	
Public place	328 (17.0)	271 (19.2)	37 (12.2)	20 (9.4)	
Street	254 (13.2)	204 (14.4)	24 (7.9)	26 (12.3)	
Witnessed by EMS	230 (11.9)	142 (10.0)	61 (20.1)	27 (12.7)	
Others	126 (6.5)	82 (5.8)	12 (4.0)	32 (15.1)	
Witnessed cardiac arrest	1,474 (76.6)	1,101 (78.1)	242 (79.6)	131 (62.4)	<0.001
Bystander CPR^b^	1,074 (56.6)	791 (56.7)	191 (64.3)	92 (44.9)	<0.001
Initial cardiac rhythm					<0.001
VF	1190 (62.1)	1010 (71.8)	75 (24.8)	105 (50.7)	
VT	37 (1.9)	28 (2.0)	4 (1.3)	5 (2.4)	
PEA	501 (26.2)	265 (18.9)	180 (59.4)	56 (27.1)	
Asystole	188 (9.8)	103 (7.3)	44 (14.5)	41 (19.8)	
Albumin, mg/dL	3.1 ± 0.7	3.1 ± 0.7	2.9 ± 0.8	3.1 ± 0.8	<0.001
Albumin <4 mg/dL^b^	1,586 (89.5)	1,177 (89.3)	248 (92.5)	161 (86.6)	0.11
eGFR, mL/min/1.73 m^2^	52.0 ± 21.9	51.4 ± 18.7	51.4 ± 27.4	57.4 ± 30.6	0.042
eGFR <60 mL/min/1.73 m^2^^a^	1,331 (71.2)	1,003 (72.9)	199 (69.6)	129 (62.3)	0.006
CRP, mg/dL	1.0 ± 3.2	0.7 ± 0.1	2.1 ± 0.2	1.6 ± 0.2	<0.001
CRP *≥*5 mg/dL^a^	83 (4.6)	39 (2.9)	28 (10.1)	16 (8.0)	<0.001
White blood cells/μL	10,176 ± 4,738	10,506 ± 4,660	9,790 ± 4,816	8,535 ± 4,782	<0.001
Hemoglobin, g/dL	12.5 ± 2.9	12.8 ± 2.6	11.5 ± 3.2	11.7 ± 3.3	<0.001
Hemoglobin <11 g/dL^a^	501 (26.8)	308 (22.5)	119 (41.3)	74 (35.6)	<0.001
Platelet ×10^4^/μL	15.3 ± 7.4	15.5 ± 7.1	14.4 ± 7.6	15.1 ± 9.8	0.015
Platelet <10 × 10^4^/μL^a^	403 (21.6)	267 (19.5)	80 (27.8)	56 (27.1)	0.001
Fibrinogen, mg/dL	226.6 ± 107.0	228.4 ± 94.4	213.4 ± 138.1	233.5 ± 128.9	<0.001
Fibrinogen <200 mg/dL^b^	705 (41.7)	488 (39.8)	139 (51.5)	78 (39.8)	0.002
APTT, s	46.0 (34.4-84.6)	45.0 (33.9-83.2)	53.5 (38.6-90.2)	45.7 (35.0-93.0)	0.008
APTT ≥60 s^b^	652 (36.8)	457 (35.2)	123 (44.9)	72 (36.6)	0.010
D-dimer, μg/mL	13.2 (5.2-30.8)	12.3 (5.0-28.2)	23.8 (9.6-68.4)	8.9 (2.8-27.1)	<0.001
D-dimer ≥5 μg/mL^a^	1,282 (75.6)	925 (75.2)	241 (86.7)	116 (61.7)	<0.001
Lactate, mmol/L	12.9 ± 4.3	13.1 ± 4.1	12.8 ± 4.4	11.4 ± 5.2	<0.001
Lactate >5 mmol/L^a^	1,634 (95.7)	1,235 (97.2)	239 (95.2)	160 (86.5)	<0.001
ECMO cannulation place					<0.001
Emergency room	1,250 (65.1)	887 (63.0)	194 (64.2)	169 (79.7)	
Catheter laboratory^a^	668 (34.8)	517 (36.7)	108 (35.8)	43 (20.3)	
Others	3 (0.2)	3 (0.2)	0 (0.0)	0 (0.0)	
Institution^a^					
Q1	1,074 (55.5)	750 (52.9)	197 (64.6)	127 (59.6)	<0.001
Q2	484 (25.0)	369 (26.0)	67 (22.0)	48 (22.5)	0.22
Q3	259 (13.4)	197 (13.9)	29 (9.5)	33 (15.5)	0.078
Q4	118 (6.1)	101 (7.1)	12 (3.9)	5 (2.4)	0.006
Coronary angiography	1,364 (70.5)	1,180 (83.3)	119 (39.0)	65 (30.5)	<0.001
Percutaneous catheter intervention	766 (40.8)	745 (54.0)	13 (4.5)	8 (3.9)	<0.001
IABP insertion^a^	1,099 (56.9)	1,000 (70.7)	60 (19.9)	39 (18.3)	<0.001
Temporary pacemaker	105 (5.8)	95 (7.2)	6 (2.1)	4 (2.0)	<0.001
CRRT	241 (12.5)	199 (14.0)	22 (7.2)	20 (9.4)	0.002
Length of ICU stay, d	3 (1-10)	4 (1-11)	1 (1-5)	2 (1-5)	<0.001
Length of hospital stay, d	3 (1-18)	4 (1-22)	2 (1-6)	2 (1-10)	<0.001
Survival at hospital discharge	500 (25.8)	404 (28.5)	45 (14.8)	51 (23.9)	<0.001

Values are mean ± SD or n (%).

APTT = activated partial thromboplastin time; CPR = cardiopulmonary resuscitation; CRP = C-reactive protein; CRRT = continuous renal replacement therapy; ECMO = extracorporeal membrane oxygenation; eGFR = estimated glomerular filtration rate; EMS = emergency medical service; IABP = intra-aortic balloon pumping; ICU = intensive care unit; PEA = pulseless electrical activity; VF = ventricular fibrillation; VT = ventricular tachycardia.

a Covariables used in the multivariate analysis.

b Covariates added in the sensitivity analysis.

As a sensitivity analysis complementary to Cox models, we estimated restricted mean survival time (RMST) at prespecified horizons (τ = 28, 7, and 3 days) using propensity score overlap weighting targeting the average treatment effect in the overlap population. Propensity scores were estimated with multinomial logistic regression for OHCA causes (3 levels) and logistic regression for IABP (binary), including the same prespecified covariates as in the Cox models; analyses were conducted on complete cases. For each group, weighted survival curves were obtained with survfit using the overlap weights, and RMST was computed as the area under the weighted event-free survival curve up to τ. Pairwise ΔRMST (difference in RMST) and 95% CIs were obtained from a nonparametric percentile bootstrap (B = 500) with re-estimation of the propensity scores and weights at each replicate. No weighted log-rank test was performed for overlap-weighted curves; inference relied on ΔRMST with bootstrap CIs. For OHCA causes and IABP, the prespecified primary exposure, we applied a fixed-sequence procedure across τ = 28, 7, and 3 days (2-sided α = 0.05 was applied at each step; downstream horizons were formally tested only if the preceding test was significant); otherwise, ΔRMSTs were reported descriptively. Covariate balance after weighting was checked using absolute standardized mean differences (threshold 0.10) and overlap plots.

All *P* values were 2-sided, and *P <* 0.05 was considered significant. Missing values were not imputed and were handled as missing values. All variables used in the multivariate analysis and outcome measures assessed in this analysis were prespecified in the registry. Statistical analyses were performed using JMP software (version 18.0, SAS Institute Japan), and R version 4.3.3 (R Foundation for Statistical Computing). The primary packages included survival, WeightIt, cobalt, and survminer.

## Results

**Figure 1:**
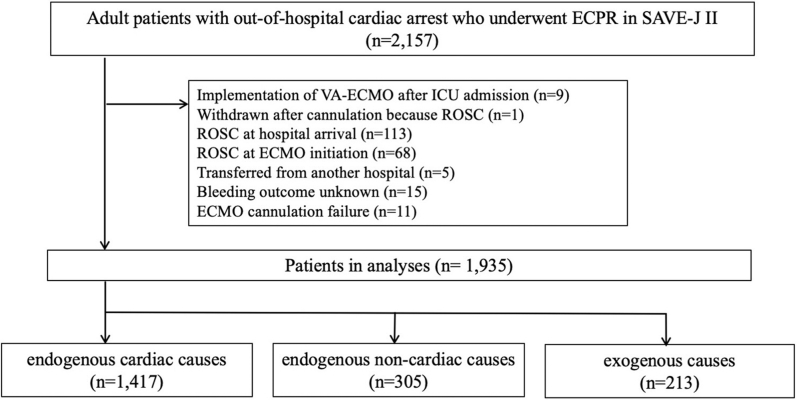
Flowchart Depicting Patient Screening and Selection Patients underwent extracorporeal cardiopulmonary resuscitation (ECPR) for out-of-hospital cardiac arrest from 2013 to 2018 were stratified into endogenous cardiac, endogenous noncardiac, and exogenous causes groups, according to underlying causes of out-of-hospital cardiac arrest. ICU = intensive care unit; ROSC = return of spontaneous circulation; VA-ECMO = venoarterial extracorporeal membrane oxygenation.

A participant selection flowchart is shown in Figure 1. Among the 2,157 adult patients with OHCA who received ECPR in SAVE-J II, 1,935 patients were included in this study: 1,417 had endogenous cardiac causes, 305 had endogenous noncardiac causes, and 213 had exogenous causes. The median follow-up period was 3 days (Q1-Q3: 1-18 days), and 36 days (IQR: 21-55 days) for survivors.

The mean age was 58.9 ± 14.0 years, and 1,597 patients (82.5%) were men (Table 1). Despite being younger than those in the endogenous noncardiac or exogenous stratum, participants in the endogenous cardiac stratum had more cardiac risk factors and comorbidities, such as hypertension, diabetes mellitus, dyslipidemia, or history of cardiovascular diseases; had antithrombotic agents prescribed more frequently before admission; and more frequently underwent additional invasive treatment, including coronary angiography, percutaneous catheter intervention, IABP insertion, and placement of temporary pacemakers.

**Figure 2:**
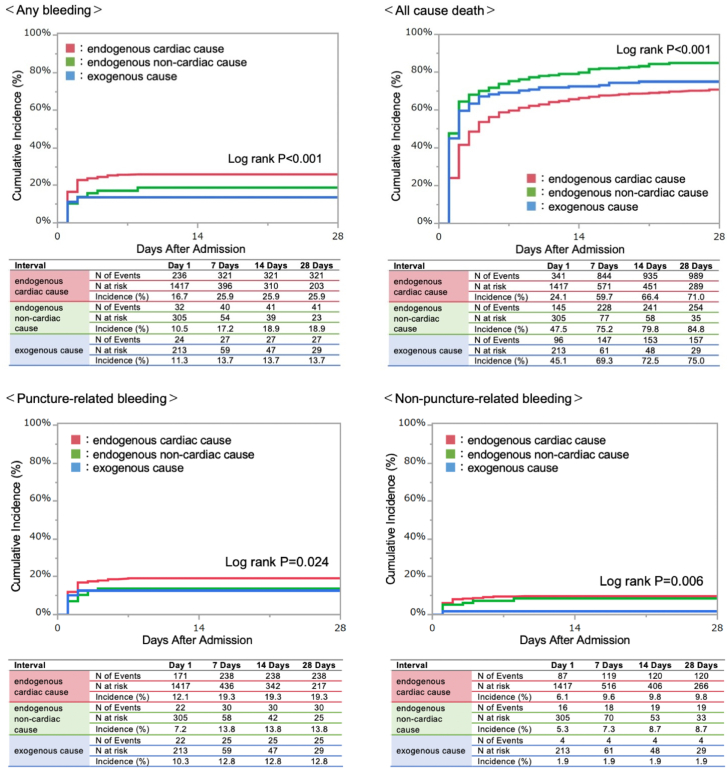
Cumulative Incidences of the Clinical Outcomes The 30-day cumulative incidence of any bleeding significantly differed among the endogenous cardiac, endogenous noncardiac, and exogenous causes groups.

Among the current study population, 389 patients (20.1%) developed bleeding complications during hospitalization. Most of the bleeding events occurred within a week after ECPR (median 2 days [Q1-Q3: 1-8 days]). The 30-day cumulative incidence of any bleeding was highest in the patients with endogenous cardiac causes, followed by endogenous noncardiac and exogenous causes (endogenous cardiac: n = 321 [25.9%]; endogenous noncardiac: n = 41 [18.9%]; and exogenous: n = 27 [13.7%]; *P* < 0.001) (Figure 2). The incidence of procedure-related bleeding was higher in patients with endogenous cardiac causes (endogenous cardiac: n = 238 [19.3%]; endogenous noncardiac: n = 30 [13.8%]; and exogenous: 25 [12.8%]; *P =* 0.024) whereas that of nonprocedure-related bleeding was higher in patients with endogenous cardiac and noncardiac causes (endogenous cardiac: n = 120 [9.8%]; endogenous noncardiac: n = 19 [8.7%]; and exogenous: n = 4 [1.9%]; *P =* 0.006). The cumulative 30-day incidence of all-cause death was higher in patients with endogenous noncardiac causes, followed by those with exogenous and endogenous cardiac causes (endogenous cardiac: n = 989 [71.0%]; endogenous noncardiac: n = 254 [84.8%]; and exogenous: n = 157 [75.0%]; *P* < 0.001).

In a multivariate analysis using the Cox proportional hazard model, the causes of OHCA showed no significant difference in bleeding endpoints after adjustment. However, IABP use was associated with higher risk for any bleeding (HR: 1.72; 95% CI: 1.27-2.31; *P* < 0.001) (Table 2), procedure-related bleeding (HR: 1.68, 95% CI: 1.21-2.36; *P =* 0.002) (Table 2), and nonprocedure-related bleeding (HR: 2.80; 95% CI: 1.60-5.14; *P* < 0.001) (Table 2). IABP use was associated with lower risk for all-cause death (HR: 0.55; 95% CI: 0.47-0.63; *P* < 0.001) (Table 2). Sensitivity analysis using 20 covariates confirmed the results; the causes of OHCA showed no significant difference in bleeding endpoints after adjustment, whereas IABP use was associated with higher risk for any bleeding (HR: 1.84; 95% CI: 1.33-2.53; *P* < 0.001) (Supplemental Table 1), procedure-related bleeding (HR: 1.84; 95% CI: 1.29-2.66; *P =* 0.001) (Supplemental Table 1), and nonprocedure-related bleeding (HR: 2.87; 95% CI: 1.56-5.27; *P* < 0.001) (Supplemental Table 1). Furthermore, IABP use was associated with lower risk for all-cause death (HR: 0.55; 95% CI: 0.47-0.64; *P* < 0.001) (Supplemental Table 1). Age ≥75 years was associated with high risk of any bleeding and procedure-related bleeding, whereas there was no significant difference for nonprocedure-related bleeding (Table 2, Supplemental Table 1). Participating institute category Q4 was associated with a higher risk of any bleeding and nonprocedure-related bleeding without no apparent difference for the procedure-related bleeding relative to the category Q1 (Table 2, Supplemental Table 1).

**Table 2:** Potential Risk Factors for Bleeding and All-Cause Death

	Any Bleeding		Procedure-Related Bleeding		Nonprocedure-Related Bleeding		All-Cause Death	
	HR (95%CI)	*P* Value	HR (95%CI)	*P* Value	HR (95%CI)	*P* Value	HR (95%CI)	*P* Value
Endogenous noncardiac stratum^a^	0.88 (0.59-1.30)	0.51	0.83 (0.52-1.27)	0.40	1.64 (0.87-2.95)	0.12	1.06 (0.88-1.26)	0.54
Exogenous stratum^a^	0.65 (0.38-1.09)	0.086	0.75 (0.42-1.26)	0.29	0.33 (0.05-1.09)	0.72	0.98 (0.79-1.21)	0.86
IABP use	1.72 (1.27-2.31)	<0.001	1.68 (1.21-2.36)	0.002	2.80 (1.60-5.14)	<0.001	0.55 (0.47-0.63)	<0.001
Age ≥75 y	1.56 (1.13-2.14)	0.010	1.58 (1.08-2.24)	0.018	1.58 (0.87-2.68)	0.12	1.22 (1.01-1.45)	0.036
eGFR <60 mL/min/1.73 m^2^	0.79 (0.62-1.01)	0.067	0.75 (0.57-0.99)	0.042	0.95 (0.62-1.50)	0.82	1.43 (1.24-1.66)	<0.001
Hemoglobin <11 g/dL	0.94 (0.71-1.23)	0.63	0.99 (0.72-1.34)	0.94	0.72 (0.43-1.16)	0.18	1.13 (0.98-1.30)	0.089
Platelet <10 × 10^4^/μL	1.25 (0.95-1.64)	0.12	1.19 (0.86-1.61)	0.28	1.64 (1.04-2.53)	0.035	1.22 (1.05-1.41)	0.009
D-dimer ≥5 mg/dL	1.30 (0.97-1.73)	0.074	1.18 (0.87-1.65)	0.29	1.84 (1.08-3.35)	0.024	1.19 (1.02-1.38)	0.028
CRP *≥*5 mg/dL	0.77 (0.41-1.46)	0.41	0.79 (0.36-1.51)	0.51	0.68 (0.17-1.85)	0.50	1.04 (0.79-1.34)	0.79
Lactate >5 mmol/L	1.47 (0.69-1.64)	0.29	1.43 (0.68-3.67)	0.37	1.40 (0.43-8.56)	0.63	1.39 (1.01-1.97)	0.044
Antithrombotic agents	0.86 (0.61-1.22)	0.39	0.86 (0.57-1.25)	0.43	1.04 (0.57-1.77)	0.90	0.79 (0.65-0.94)	0.010
Catheter laboratory puncture	1.26 (0.99-1.61)	0.064	1.14 (0.86-1.65)	0.37	1.27 (0.83-1.94)	0.27	0.96 (0.84-1.10)	0.59
Institute Q2^b^	1.04 (0.79-1.36)	0.80	1.00 (0.73-1.35)	0.99	1.23 (0.75-1.97)	0.40	1.02 (0.88-1.18)	0.79
Institute Q3^b^	1.15 (0.79-1.67)	0.46	1.11 (0.71-1.67)	0.65	1.79 (0.96-3.16)	0.066	1.16 (0.95-1.42)	0.15
Institute Q4^b^	1.67 (1.11-2.52)	0.020	1.44 (0.86-2.30)	0.16	2.36 (1.18-4.37)	0.018	1.10 (0.83-1.42)	0.50

Abbreviations as in Table 1.

a Risks relative to the endogenous cardiac stratum.

b Risks relative to the reference category (Institute Q1).

After propensity-score overlap weighting, covariate balance was satisfactory in both the OHCA-cause and IABP models; nearly all absolute standardized mean differences were <0.10 and none exceeded 0.25 (Supplemental Figure 1). At τ = 28 days, overlap-weighted RMST differences across OHCA causes strata were small for any bleeding and all-cause death: vs cardiac causes, noncardiac showed ΔRMST −0.25 days (95% CI: −3.04 to 2.06 days) for any bleeding and −0.19 days (95% CI: −1.97 to 1.42 days) for death, while exogenous showed +1.91 days (95% CI: −0.34 to 4.23 days) for any bleeding and +0.06 days (95% CI: −1.87 to 2.17 days) for death (Table 3, Supplemental Figure 2). For procedure-related bleeding, differences were modest and imprecise (noncardiac −0.08 days [95% CI: −2.46 to 1.87]; exogenous +0.98 days, [95% CI: −1.25 to 3.21 days]). For nonprocedure-related bleeding, exogenous vs cardiac demonstrated a modest but statistically significant longer bleeding-free time (ΔRMST +0.96 days [95% CI: 0.34-1.62]), whereas noncardiac vs cardiac was −1.41 days (95% CI: −3.47 to 0.31). Using IABP as the exposure, RMST indicated shorter bleeding-free time (ΔRMST [IABP − No IABP] −2.69 days for any bleeding, −2.13 days for procedure-related, and −1.74 days for nonprocedure-related; all CIs excluding 0) and longer survival time (ΔRMST +5.58 days [95% CI: 4.26-6.77]) at 28 days (Table 3). Effects at 7 and 3 days were directionally consistent (Supplemental Tables 2 and 3). Overlap-weighted cumulative-incidence curves were concordant—showing higher bleeding but lower mortality in the IABP group (Supplemental Figure 3). These RMST findings, which do not assume proportional hazards, were concordant in direction with the Cox models and support the robustness of the main results.

**Table 3:** Restricted Mean Survival Time Analysis for Bleeding and All-Cause Death

Outcome	Comparison Group^a^	ΔRMST (d)	95% CI	RMST Cardiac	RMST Noncardiac	RMST Exogenous
Any bleeding	Noncardiac	−0.25	−3.04 to 2.06	22.74	22.49	24.66
	Exogenous	1.91	−0.34 to 4.23	22.74	22.49	24.66
Procedure-related bleeding	Noncardiac	−0.08	−2.46 to 1.87	23.70	23.62	24.68
	Exogenous	0.98	−1.25 to 3.21	23.70	23.62	24.68
Nonprocedure-related bleeding	Noncardiac	−1.41	−3.47 to 0.31	26.70	25.28	27.66
	Exogenous	0.96	0.34-1.62	26.70	25.28	27.66
All-cause death	Noncardiac	−0.19	−1.97 to 1.42	8.05	7.86	8.11
	Exogenous	0.06	−1.87 to 2.17	8.05	7.86	8.11

	Comparison Group^b^	ΔRMST (d)	95% CI	RMST IABP Use	RMST No IABP
Any bleeding	IABP use	−2.69	−4.29 to −1.11	20.89	23.58
Procedure-related bleeding	IABP use	−2.13	−3.53 to −0.66	22.40	24.54
Nonprocedure-related bleeding	IABP use	−1.74	−2.71 to −0.82	25.26	26.99
All-cause death	IABP use	5.58	4.26-6.77	12.63	7.04

a Reference, cardiac stratum.

b Reference, no intra-aortic balloon pumping (IABP) group. ΔRestricted mean survival time (RMST) = RMST (Comparison) – RMST (Reference). Positive values indicate longer mean event-free survival in the comparison group.

## Discussion

To our knowledge, the SAVE-J II study is among the largest studies to evaluate hemorrhagic complications and their predictors in patients who underwent ECPR for OHCA. From this large-scale registry, the main findings of the current analysis were as follows: 1) approximately one-fifth of patients with OHCA experience bleeding complications within a few days after ECPR, and the incidences were different among the causes of OHCA; 2) although the cause of OHCA was not an independent predictor of bleeding, the incidence of bleeding was high in the endogenous cardiac causes group; and 3) additional IABP use on VA-ECMO was an independent risk factor for bleeding, regardless of the types of hemorrhagic complications (Central Illustration). In addition, adjacent IABP use was associated with lower risks for all-cause death.

**Central Illustration:**
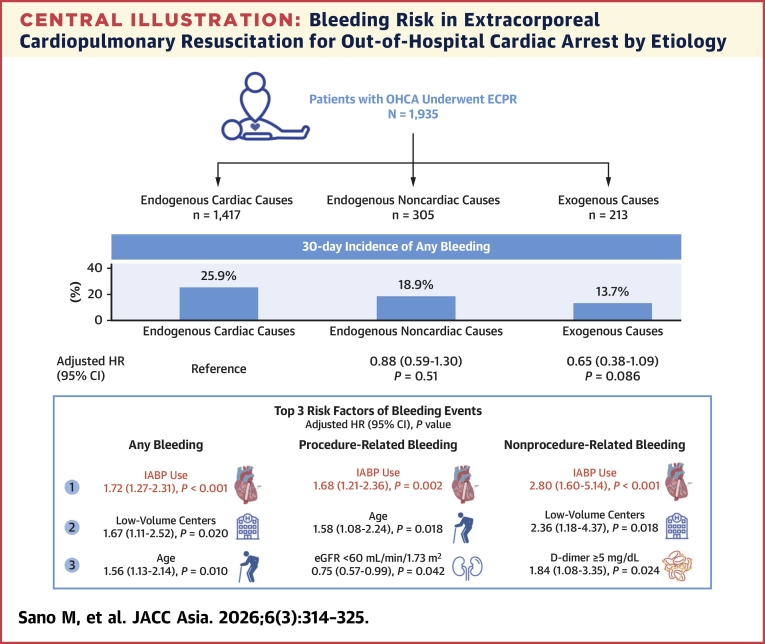
Bleeding Risk in Extracorporeal Cardiopulmonary Resuscitation for Out-of-Hospital Cardiac Arrest by Etiology The incidence of clinically relevant bleeding varied depending on the underlying causes of cardiac arrest. The incidence of bleeding was lowest for the exogenous stratum, followed by the endogenous noncardiac stratum, and highest for the endogenous cardiac stratum. Intra-aortic balloon pumping (IABP) insertion was a risk factor for hemorrhagic complications in both procedure- and nonprocedure-related bleeding. ECPR = extracorporeal cardiopulmonary resuscitation; eGFR = estimated glomerular filtration rate; OHCA = out-of-hospital cardiac arrest.

Bleeding after ECPR is one of the most common clinical problems, regardless of the cause of OHCA. Although there are differences in the study design or the bleeding definition, previous reports have shown varying rates of bleeding complications in post-ECPR patients, ranging from 8% to 70%.^9^^,^^11^, ^12^, ^13^, ^14^, ^15^ The observed incidence of bleeding was 20.1% in this study. Nevertheless, the definition of bleeding here may underestimate minor bleeding events, but it is likely to reflect clinically significant bleeding. We also observed that the incidence and risk of clinically relevant bleeding may vary depending on the underlying causes. The incidence of bleeding was lowest for the exogenous stratum (13.7%), followed by the endogenous noncardiac stratum (18.9%), and highest for the endogenous cardiac stratum (25.9%). Although OHCA causes were not independent predictors of bleeding, the underlying conditions would be beneficial in stratifying bleeding risk in patients who underwent ECPR. Because the number of noncardiogenic OHCAs was relatively small, additional data are needed to analyze their predictors. Furthermore, the bleeding occurred within several days after ECMO insertion in all strata for OHCA causes. As bleeding begets bleeding, timely treatment or an approach to reduce bleeding is crucial in managing patients after ECPR. Considering the acceptable stratification performance for bleeding risk using the OHCA causes, it would be helpful to manage patients after ECPR, taking the cause of cardiac arrest in mind.

Previous reports stated that the most frequent bleeding site in patients with ECPR was the cannulation site.^14^ For VA-ECMO, typical draining cannulas range from 19- to 25-F, and returning cannulas are 15- to 17-F.^16^ The ECMO support using large cannulas enables us to obtain high blood flow; however, they are prone to causing vascular injury. In the ECPR setting, the introduction of cardiac support is challenging because of the absence of a palpable pulse, and adequate assessment of other combined status is often unavailable. In our study, procedure-related bleeding was more common than nonprocedure-related bleeding and occurred more frequently in the endogenous cardiac group (procedure-related: 12.8%-19.3%, nonprocedure-related: 1.9%-9.8%) (Figure 2). Additional invasive treatment, IABP insertion, was an independent predictor of bleeding. The trend resembles the previous analysis for the patients who underwent percutaneous left ventricular assist device treatment.^10^ In the current cohort, older age was associated with risk of bleeding, which is in line with the previous percutaneous coronary intervention studies.^17^ As mentioned in the previous report from the same registry, a low platelet count would help predict a bleeding risk.^9^

Nonprocedure-related bleeding is another concern in patients with OHCA who undergo ECPR. Moreover, bleeding events resulted from CPR-related injuries, including sternal or rib fractures, pulmonary contusion, blunt cardiac injury, intraabdominal injury, and pneumothorax.^13^ A single-center study reported that 27% of ECPR patients required urgent surgical intervention for traumatic injury from CPR.^4^ In this analysis, nonprocedure-related bleeding occurred among 1.9% to 9.8% of patients who underwent ECPR, and the incidence differed among the OHCA causes. The bleeding definition focused on relatively severe bleeding; however, many patients experienced severe nonprocedure-related bleeding after ECPR. After adjustment, IABP use, low-volume centers, and D-dimer of over 5 mg/dL were independent risk factors for nonprocedure-related bleeding. IABP use consistently remained the risk for nonprocedure-related bleeding. This might result from additional invasive treatment including percutaneous catheter intervention or IABP insertion and concomitant disseminated intravascular coagulation, or differences in management after ECPR. Patients after cardiac arrest sometimes have complications such as coagulopathy associated with hypothermia, metabolic acidosis, and disseminated intravascular coagulation.^18^, ^19^, ^20^, ^21^ Risk factors of post-ECPR bleeding included decreasing levels of fibrinogen and higher levels of D-dimer on admission, which suggested hyperfibrinolysis may be related to bleeding.^14^^,^^22^ However, these factors were not identified as independent predictive values of bleeding events in our study.

The increased bleeding risk associated with IABP use may be related to several factors: 1) the additional vascular access site; 2) the need for more intensive anticoagulation with multiple devices; and 3) potential mechanical interaction between the IABP and ECMO flow that affects local hemodynamics at cannulation sites. In the present study, IABP use was significantly associated with bleeding complications both for procedure- and nonprocedure-related bleeding (Table 2). Despite the paucity of studies of the bleeding risk associated with IABP use in ECPR, IABP support for acute myocardial infarction patients with VA-ECMO was associated with an increased risk of major bleeding.^23^ In contrast, concomitant use of VA-ECMO and IABP was associated with improved mortality and neurological outcome in OHCA patients who underwent ECPR.^24^, ^25^, ^26^ During peripheral VA-ECMO, the arterial perfusion is retrograde and the increasing afterload on the left ventricle may lead to pulmonary edema. Thus, IABP has been considered to provide left ventricular unloading in VA-ECMO. Diastolic augmentation during IABP has been shown to increase the mean arterial pressure.^27^ In the current analysis, although the IABP use among the strata were significantly different from 18% to 71%, IABP use significantly reduced mortality risk after adjustment. It should be interpreted cautiously because of potential selection bias in registry data; however, IABP use might reduce mortality risk instead of bleeding risk in OHCA patients undergoing ECPR.

Finally, this study primarily analyzed Japanese individuals, who have a paradoxical relationship with bleeding and thrombotic risk, and have the unique drug administration dose required for approval.^28^ Asian populations including Japanese have been reported to be at a higher risk of bleeding.^28^^,^^29^ Some reports have consistently shown that bleeding complications are associated with increased mortality following ECPR.^6^, ^7^, ^8^^,^^30^ Given the higher risk of bleeding complications in patients who underwent ECPR than in patients with general coronary disease, careful attention to hemorrhagic complications is warranted, and the evaluation for bleeding risks specifically in Asian patients in this study holds significant clinical relevance. The combination of ultrasound- and fluoroscopy-guided cannulation may reduce the incidence of cannulation-related complications. However, we could not assess the influence of echo-guided puncture because of the extent of missing data.^31^ Optimal anticoagulation therapy or management during ECMO support is another critical point in reducing bleeding events. Systemic anticoagulation with unfractionated heparin is essential to prevent thrombosis in VA-ECMO, which can predispose to easy bleeding.^30^ Generally, the therapeutic anticoagulation range for ECMO is an activated clotting time (ACT) of 180 to 200 s.^32^ However, Kim et al^33^ reported that maintaining the ACT lower than the conventional value (<160 s) did not significantly increase the risk of thromboembolism, weaning failure, or mortality during ECPR management. A low ACT target would be a reasonable option for patients with high bleeding risk.^33^ Mechanical support devices cannot be excluded from the risk of acquired von Willebrand disease because of the exposure of the von Willebrand factor to the nonphysiological shear stress that occurs in the blood pump or from platelet consumption in the circuit. Furthermore, appropriate transfusion of platelets or plasma to supplement excess usage during mechanical support use is crucial. Additionally, it is worth noting that the dosage of approved antithrombotic drugs in Japan differs from those in other regions.^28^ Considering the higher risk of bleeding in Asian patients, titrated antithrombotic drug doses approved in Japan might be a reasonable option. Actually, the use of oral antithrombotic agents had a neutral effect on bleeding in the current analysis. To date, there are no established strategies to reduce bleeding complications in ECPR. Further multinational studies incorporating diverse ethnic groups are essential to advance our understanding, and detailed analyses of the current status and complications associated with ECPR are warranted.

### Study limitations

First, this observational registry data included heterogeneous patient populations without standardized treatment protocols across participating centers. Therefore, we cannot exclude the effect of unmeasured confounding factors that potentially affected our results. Second, because of the retrospective nature of current analysis, we could not use standardized bleeding criteria such as Extracorporeal Life Support Organization,^34^ Bleeding Academic Research Consortium (BARC),^35^ or Global Use of Strategies to Open Occluded Arteries (GUSTO)^36^ criteria. However, our bleeding definition corresponds to GUSTO bleeding criteria of moderate or higher and BARC bleeding criteria of type 3 or higher. Although we might have underestimated minor bleeding, we have evaluated bleeding that requires critical medical intervention in post-ECPR management. Third, the current data set did not include patients with percutaneous ventricular assist devices (PVAD) because they were unavailable during the study period in Japan. Nonetheless, PVAD use has been increasing, and the associated bleeding risk has been reported to be higher than with IABP.^37^ Therefore, additional evaluation of the bleeding risk of PVAD use in ECPR would be needed. Fourth, although we adjusted for clinically relevant variables, some potentially valuable factors, such as details of anticoagulation management (eg, ACT targets), timing and volume of blood product administration, and specific technical aspects of ECMO cannulation, were unavailable in our data set. In addition, we used 11 clinically selected risk-adjusting covariates to minimize potential bias; however, there might exist inevitable selection bias. As a sensitivity analysis, we added additional sensitivity using 20 covariates to confirm the results; however, we need other validation analyses to confirm the results. As a prespecified analysis, we primarily used visual inspection of log(−log[survival]) vs log(time) plots, stratified by each variable; however, there is a concern of invalid assumption of proportionality of hazards in each covariate. To confirm the result of the current analysis, we performed sensitivity analysis using propensity score and RMST analysis, which is nonparametric, free from proportionality of hazards of covariates. As a result, time-scale robust RMST analyses at prespecified horizons provided consistent confirmation with the Cox proportional hazards model. Fifth, the definitions of procedure- or nonprocedure-related bleeding were prespecified before the data collection; however, the definition might include classification bias. Finally, the association between IABP use and improved survival should be interpreted cautiously because of the potential selection bias inherent in the use of registry data.

## Conclusions

Despite the differences in the predictors for bleeding according to the bleeding type, additional mechanical circulatory support use was a strong predictor of bleeding in patients who underwent ECPR. Vigilant monitoring for post-ECPR bleeding complications is crucial in OHCA patients, especially those with additional circulatory support devices.

## Funding Support and Author Disclosures

The authors have reported that they have no relationships relevant to the contents of this paper to disclose.
