# Supplementary material for: Predictors of Bleeding Complications After Extracorporeal Cardiopulmonary Resuscitation: Insights From the SAVE-J II Study
Source: JACC Asia. 2025 Dec 19;6(3):314–25. doi: 10.1016/j.jacasi.2025.09.027 (PMC12959310; doi:10.1016/j.jacasi.2025.09.027)
Supplement: Supplemental Material [file mmc1.docx]

STROBE Statement—checklist of items that should be included in reports of observational studies

|  | Item No. | Recommendation | Page  No. | Relevant text from manuscript |
| --- | --- | --- | --- | --- |
| **Title and abstract** | 1 | (*a*) Indicate the study’s design with a commonly used term in the title or the abstract | 1 | Predictors of Bleeding Complications After Extracorporeal Cardiopulmonary Resuscitation: **Insights from the SAVE-J II Registry** |
|  |  | (*b*) Provide in the abstract an informative and balanced summary of what was done and what was found | 5,6 | **Background:** Extracorporeal cardiopulmonary resuscitation (ECPR), an emerging resuscitative therapy following refractory cardiac arrests, is associated with hemorrhagic complications that potentially affect patient outcomes.  **Objectives:** This study evaluated the risks and predictors of hemorrhagic complications among patients who underwent ECPR for out-of-hospital cardiac arrest (OHCA) from different causes.  **Methods:** Using the SAVE-J II registry, we analyzed multicentric data of patients who underwent ECPR for OHCA from 2013 to 2018 in Japan. Based on the causes of OHCA, the participants were stratified into endogenous cardiac, endogenous non-cardiac, and exogenous groups. The primary outcome was any bleeding.  **Results:** Among 1,935 patients, 1,417, 305, and 213 had endogenous cardiac, endogenous non-cardiac, and exogenous causes, respectively. For survivors, the median follow-up period was 36 days and most of the bleeding events occurred within one week post-ECPR. The 30-day cumulative incidence of any bleeding significantly differed among the three groups (endogenous cardiac: 321 patients [25.9%]; endogenous non-cardiac: 41 patients [18.9%]; and exogenous: 27 patients [13.7%], P<0.001). However, the risks for bleeding complications did not differ between the causes of OHCA after adjustment for confounders. Intra-aortic balloon pumping (IABP) use was associated with higher risks of bleedings and lower risk for all-cause death.  **Conclusions:** Underlying causes of OHCA did not significantly impact adjusted bleeding risks. IABP use was independently associated with higher bleeding risks and lower mortality, although this warrants cautious interpretation owing to a potential selection bias. Vigilant monitoring for bleeding complications is crucial in ECPR patients, especially in those with additional circulatory support devices. |
| Introduction | | | |  |
| Background/rationale | 2 | Explain the scientific background and rationale for the investigation being reported | 7, 8 | With the increasing number of patients experiencing out-of-hospital cardiac arrest (OHCA), extracorporeal cardiopulmonary resuscitation (ECPR) has emerged as a valuable intervention for patients with refractory cardiac arrest.1 ECPR with venoarterial extracorporeal membrane oxygenation (VA-ECMO) support in patients with OHCA ensures sufficient organ perfusion and oxygen supply. Therefore, the adaptation of ECPR is expected to improve survival rates or neurological outcomes of patients with OHCA compared with treatment with conventional cardiopulmonary resuscitation (CPR) only.2-5 However, ECPR is a highly invasive intervention that is associated with various complications, including thrombosis, hemolysis, limb ischemia, infection, and bleeding events.3  Among them, bleeding is the most common complication and associated with increase mortality rates.6-8 The multifactorial causation of bleeding risk during ECPR includes the requirement for anticoagulation, insertion of large cannulas, CPR-related trauma, and coagulopathy associated with postcardiac arrest syndrome. The use of additional mechanical circulatory support devices, such as intra-aortic balloon pump (IABP), could reduce the cardiac afterload and cardiac burden although it could adversely increase the bleeding risk. The underlying causes of OHCA are varied and may be attributed to the patient’s clinical outcomes. Despite the increasing use of ECPR, there remains a critical knowledge gap regarding the influence of different underlying causes of cardiac arrest on bleeding risk. Understanding the factors that predispose patients to bleeding during ECPR is crucial for optimizing patient management and improving outcomes. However, comprehensive data on associated risk factors in ECPR patients remain scarce. |
| Objectives | 3 | State specific objectives, including any prespecified hypotheses | 7-9 | Among them, bleeding is the most common complication and associated with increase mortality rates.6-8 The multifactorial causation of bleeding risk during ECPR includes the requirement for anticoagulation, insertion of large cannulas, CPR-related trauma, and coagulopathy associated with postcardiac arrest syndrome. The use of additional mechanical circulatory support devices, such as intra-aortic balloon pump (IABP), could reduce the cardiac afterload and cardiac burden although it could adversely increase the bleeding risk. The underlying causes of OHCA are varied and may be attributed to the patient’s clinical outcomes. Despite the increasing use of ECPR, there remains a critical knowledge gap regarding the influence of different underlying causes of cardiac arrest on bleeding risk. Understanding the factors that predispose patients to bleeding during ECPR is crucial for optimizing patient management and improving outcomes. However, comprehensive data on associated risk factors in ECPR patients remain scarce.  From the SAVE-J II study database, an analysis was performed to identify the risk factors for bleeding during the first day of admission and to comprehensively describe details of bleeding during hospitalization in patients with cardiogenic OHCA undergoing ECPR.9 They reported that 22.1% experienced bleeding predominantly at the cannulation site and that platelet count of below 100,000/μL on admission was an independent risk factor for early bleeding events. As shown in this study, we frequently experience cannulation-site severe bleeding in patients using large bore devices like ECMO soon after admission. On the other hand, we also experience severe bleeding after the first day of admission and non-procedure-related bleeding. We reported additional mechanical circulatory device use would increase bleeding events in patients with cardiogenic shock treated by percutaneous ventricular assist device.10 To assess longer-term bleeding events and the effect of additional cardiac support devices on bleeding, including patients with non-cardiogenic OHCA, we performed a current analysis.  We hypothesized that bleeding risk in patients with ECPR would differ among the underlying causes of OHCA or the use of mechanical circulatory support devices. In this study, we aimed to evaluate the incidence and predictors of bleeding complications in patients undergoing ECPR, with specific attention to OHCA etiology and concurrent use of mechanical circulatory support. Through a better understanding of these relationships, we sought to identify opportunities for improving risk stratification and patient outcomes. |
| Methods | | | |  |
| Study design | 4 | Present key elements of study design early in the paper | 9, 10 | Study Design  The SAVE-J II study is a multicenter retrospective registry conducted in Japan, with 36 participating institutions11, and includes patients aged 18 years or older who were admitted to the emergency department with OHCA and received ECPR, between January 2013 and December 2018. The exclusion criteria for the current analysis were as follows: patients who received VA-ECMO after intensive care unit (ICU) admission, were withdrawn after cannulation due to the return of spontaneous circulation (ROSC), achieved ROSC at hospital arrival and ECMO initiation, were transferred from other hospitals, and had unknown outcomes, including bleeding complications.  The patients were stratified into three groups based on the underlying causes of OHCA as follows: endogenous cardiac, endogenous non-cardiac, and exogenous. The primary outcome was any bleeding. The secondary outcomes included bleeding related to the procedure, at cannulation sites and non-procedure-related bleeding. Any bleeding was defined as cases requiring blood transfusion, interventional radiology or surgical hemostasis. Bleeding events were classified into two prespecified categories: procedure-related bleeding and non- procedure-related bleeding. In detail, procedure-related bleeding included bleeding at cannulation sites, retroperitoneum, and puncture sites excluding ECMO cannulation, and non-procedure-related bleeding was comprised of bleeding at the brain, upper airway, chest (including mediastinal bleeding, hemothorax, pulmonary hemorrhage, etc.), abdomen (including gastrointestinal tract bleeding, liver, spleen, and abdominal cavity), and other sites. Blood transfusion was defined as the administration of packed red blood cells beyond what was required for routine ECMO circuit maintenance, such as consumption through ECMO device. |
| Setting | 5 | Describe the setting, locations, and relevant dates, including periods of recruitment, exposure, follow-up, and data collection | 10 | The current analysis was performed as a part of the data from SAVE-J II registry, which was registered at the University Hospital Medical Information Network Clinical Trials Registry and the Japanese Clinical Trial Registry (registration number: UMIN000036490), approved by the institutional review board of Kagawa University (approval number: 2018–110) and each participating institution, including Kobe City Medical Center General Hospital (approval number: zn200304. Informed consent from the patient was waived owing to the retrospective nature of the study design, and all procedures were performed in accordance with the ethical standards of the review board of Kobe City Medical Center General Hospital on human experimentation and with the Declaration of Helsinki of 1975. |
| Participants | 6 | (*a*) *Cohort study*—Give the eligibility criteria, and the sources and methods of selection of participants. Describe methods of follow-up  *Case-control study*—Give the eligibility criteria, and the sources and methods of case ascertainment and control selection. Give the rationale for the choice of cases and controls  *Cross-sectional study*—Give the eligibility criteria, and the sources and methods of selection of participants | 9  Figure 1 | The SAVE-J II study is a multicenter retrospective registry conducted in Japan, with 36 participating institutions10, and includes patients aged 18 years or older who were admitted to the emergency department with OHCA and received ECPR, between January 2013 and December 2018. The exclusion criteria for the current analysis were as follows: patients who received VA-ECMO after intensive care unit (ICU) admission, were withdrawn after cannulation due to the return of spontaneous circulation (ROSC), achieved ROSC at hospital arrival and ECMO initiation, were transferred from other hospitals, and had unknown outcomes, including bleeding complications. |
|  |  | (*b*) *Cohort study*—For matched studies, give matching criteria and number of exposed and unexposed  *Case-control study*—For matched studies, give matching criteria and the number of controls per case | - | Not applicable |
| Variables | 7 | Clearly define all outcomes, exposures, predictors, potential confounders, and effect modifiers. Give diagnostic criteria, if applicable | 9-10,  11-13 | The patients were stratified into three groups based on the underlying causes of OHCA as follows: endogenous cardiac, endogenous non-cardiac, and exogenous. The primary outcome was any bleeding. The secondary outcomes included bleeding related to the procedure, at cannulation sites and non-procedure-related bleeding. Any bleeding was defined as cases requiring blood transfusion, interventional radiology or surgical hemostasis. Bleeding events were classified into two prespecified categories: procedure-related bleeding and non- procedure-related bleeding. In detail, procedure-related bleeding included bleeding at cannulation sites, retroperitoneum, and puncture sites excluding ECMO cannulation, and non-procedure-related bleeding was comprised of bleeding at the brain, upper airway, chest (including mediastinal bleeding, hemothorax, pulmonary hemorrhage, etc.), abdomen (including gastrointestinal tract bleeding, liver, spleen, and abdominal cavity), and other sites. Blood transfusion was defined as the administration of packed red blood cells beyond what was required for routine ECMO circuit maintenance, such as consumption through ECMO device.  Statistical analysis  Continuous variables were expressed as median with interquartile range (IQR) or mean and standard deviation and were compared using the Mann-Whitney U test or Student t test based upon their distribution. For comparisons among three groups, the Kruskal-Wallis test was used. Categorical variables were expressed as numbers and percentages and compared with the chi-square test or Fisher’s exact test as appropriate. Participant institutions were classified into four groups (institution Q1 to Q4) using quartiles in descending order of their ECPR cases. The cumulative incidences of clinical outcomes were estimated using the Kaplan-Meier method, and differences among each group were assessed using a log-rank test. The multivariable Cox proportional hazards assumptions were conducted for the primary outcome measure. We selected 11 clinically relevant risk-adjusting variables listed in Table 1, a priori: OHCA causes, IABP use, age, estimated glomerular filtration rate (eGFR), hemoglobin, platelet, C-reactive protein (CRP), lactate, use of antithrombotic agents before admission, catheter laboratory puncture, and participated institution category. These covariates were selected based on clinical relevance and prior literature, not on statistical significance in univariate analysis. We also planned a sensitivity analysis before implementing statistical analyses to confirm the robustness of our findings by adding following covariates to the model; activated partial thromboplastin time (APTT), fibrinogen, albumin, hypertension, diabetes, chronic renal failure, cardiac disease, cerebrovascular disease, bystander CPR. Proportional hazards assumptions for the risk-adjustment variables, including categorized OHCA causes, were evaluated using plots of log (time) versus log (-log(survival)) stratified by the variables and deemed acceptable.  As a sensitivity analysis complementary to Cox models, we estimated restricted mean survival time (RMST) at prespecified horizons (τ = 28, 7, and 3 days) using propensity score overlap weighting targeting the average treatment effect in the overlap population (ATO). Propensity scores were estimated with multinomial logistic regression for OHCA causes (3 levels) and logistic regression for IABP (binary), including the same prespecified covariates as in the Cox models; analyses were conducted on complete cases. For each group, weighted survival curves were obtained with survfit using the overlap weights, and RMST was computed as the area under the weighted event-free survival curve up to τ. Pairwise ΔRMST (difference in RMST) and 95% CIs were obtained from a nonparametric percentile bootstrap (B = 500) with re‑estimation of the propensity scores and weights at each replicate. No weighted log‑rank test was performed for overlap‑weighted curves; inference relied on ΔRMST with bootstrap CIs. For OHCA causes and IABP, the prespecified primary exposure, we applied a fixed‑sequence procedure across τ = 28, 7, and 3 days (two-sided α=0.05 was applied at each step; downstream horizons were formally tested only if the preceding test was significant); otherwise, ΔRMSTs were reported descriptively. Covariate balance after weighting was checked using absolute standardized mean differences (threshold 0.10) and overlap plots.  All P-values were 2-sided, and P < 0.05 was considered significant. Missing values were not imputed and were handled as missing value. All variables used in the multivariate analysis and outcome measures assessed in this analysis were prespecified in the registry. Statistical analyses were performed using JMP software (version 18.0, SAS institute Japan, Tokyo, Japan), and R (version 4.3.3, R Foundation for Statistical Computing, Vienna, Austria). The primary packages included survival, WeightIt, cobalt, and survminer. |
| Data sources/ measurement | 8* | For each variable of interest, give sources of data and details of methods of assessment (measurement). Describe comparability of assessment methods if there is more than one group | 9-10 | Study Design  The SAVE-J II study is a multicenter retrospective registry conducted in Japan, with 36 participating institutions10, and includes patients aged 18 years or older who were admitted to the emergency department with OHCA and received ECPR, between January 2013 and December 2018. The exclusion criteria for the current analysis were as follows: patients who received VA-ECMO after intensive care unit (ICU) admission, were withdrawn after cannulation due to the return of spontaneous circulation (ROSC), achieved ROSC at hospital arrival and ECMO initiation, were transferred from other hospitals, and had unknown outcomes, including bleeding complications.  The patients were stratified into three groups based on the underlying causes of OHCA as follows: endogenous cardiac, endogenous non-cardiac, and exogenous. The primary outcome was any bleeding. The secondary outcomes included bleeding related to the procedure, at cannulation sites and non-procedure-related bleeding. Any bleeding was defined as cases requiring blood transfusion, interventional radiology or surgical hemostasis. Bleeding events were classified into two prespecified categories: procedure-related bleeding and non- procedure-related bleeding. In detail, procedure-related bleeding included bleeding at cannulation sites, retroperitoneum, and puncture sites excluding ECMO cannulation, and non-procedure-related bleeding was comprised of bleeding at the brain, upper airway, chest (including mediastinal bleeding, hemothorax, pulmonary hemorrhage, etc.), abdomen (including gastrointestinal tract bleeding, liver, spleen, and abdominal cavity), and other sites. Blood transfusion was defined as the administration of packed red blood cells beyond what was required for routine ECMO circuit maintenance, such as consumption through ECMO device. |
| Bias | 9 | Describe any efforts to address potential sources of bias | 11, 12 | The multivariable Cox proportional hazards assumptions were conducted for the primary outcome measure. We selected 11 clinically relevant risk-adjusting variables listed in Table 1, a priori: OHCA causes, IABP use, age, estimated glomerular filtration rate (eGFR), hemoglobin, platelet, C-reactive protein (CRP), lactate, use of antithrombotic agents before admission, catheter laboratory puncture, and participated institution category. These covariates were selected based on clinical relevance and prior literature, not on statistical significance in univariate analysis. We also planned a sensitivity analysis before implementing statistical analyses to confirm the robustness of our findings by adding following covariates to the model; activated partial thromboplastin time (APTT), fibrinogen, albumin, hypertension, diabetes, chronic renal failure, cardiac disease, cerebrovascular disease, bystander CPR. Proportional hazards assumptions for the risk-adjustment variables, including categorized OHCA causes, were evaluated using plots of log (time) versus log (-log(survival)) stratified by the variables and deemed acceptable.  As a sensitivity analysis complementary to Cox models, we estimated restricted mean survival time (RMST) at prespecified horizons (τ = 28, 7, and 3 days) using propensity score overlap weighting targeting the average treatment effect in the overlap population (ATO). Propensity scores were estimated with multinomial logistic regression for OHCA causes (3 levels) and logistic regression for IABP (binary), including the same prespecified covariates as in the Cox models; analyses were conducted on complete cases. For each group, weighted survival curves were obtained with survfit using the overlap weights, and RMST was computed as the area under the weighted event-free survival curve up to τ. Pairwise ΔRMST (difference in RMST) and 95% CIs were obtained from a nonparametric percentile bootstrap (B = 500) with re‑estimation of the propensity scores and weights at each replicate. No weighted log‑rank test was performed for overlap‑weighted curves; inference relied on ΔRMST with bootstrap CIs. For OHCA causes and IABP, the prespecified primary exposure, we applied a fixed‑sequence procedure across τ = 28, 7, and 3 days (two-sided α=0.05 was applied at each step; downstream horizons were formally tested only if the preceding test was significant); otherwise, ΔRMSTs were reported descriptively. Covariate balance after weighting was checked using absolute standardized mean differences (threshold 0.10) and overlap plots. |
| Study size | 10 | Explain how the study size was arrived at | 9  Figure 1 | Study Design  The SAVE-J II study is a multicenter retrospective registry conducted in Japan, with 36 participating institutions10, and includes patients aged 18 years or older who were admitted to the emergency department with OHCA and received ECPR, between January 2013 and December 2018. The exclusion criteria for the current analysis were as follows: patients who received VA-ECMO after intensive care unit (ICU) admission, were withdrawn after cannulation due to the return of spontaneous circulation (ROSC), achieved ROSC at hospital arrival and ECMO initiation, were transferred from other hospitals, and had unknown outcomes, including bleeding complications. |
| Quantitative variables | 11 | Explain how quantitative variables were handled in the analyses. If applicable, describe which groupings were chosen and why | 9-10 | The patients were stratified into three groups based on the underlying causes of OHCA as follows: endogenous cardiac, endogenous non-cardiac, and exogenous. The primary outcome was any bleeding. The secondary outcomes included bleeding related to the procedure, at cannulation sites and non-procedure-related bleeding. Any bleeding was defined as cases requiring blood transfusion, interventional radiology or surgical hemostasis. Bleeding events were classified into two prespecified categories: procedure-related bleeding and non- procedure-related bleeding. In detail, procedure-related bleeding included bleeding at cannulation sites, retroperitoneum, and puncture sites excluding ECMO cannulation, and non-procedure-related bleeding was comprised of bleeding at the brain, upper airway, chest (including mediastinal bleeding, hemothorax, pulmonary hemorrhage, etc.), abdomen (including gastrointestinal tract bleeding, liver, spleen, and abdominal cavity), and other sites. Blood transfusion was defined as the administration of packed red blood cells beyond what was required for routine ECMO circuit maintenance, such as consumption through ECMO device. |

Continued on next page

| Statistical methods | 12 | (*a*) Describe all statistical methods, including those used to control for confounding | 11-13 | Statistical analysis  Continuous variables were expressed as median with interquartile range (IQR) or mean and standard deviation and were compared using the Mann-Whitney U test or Student t test based upon their distribution. For comparisons among three groups, the Kruskal-Wallis test was used. Categorical variables were expressed as numbers and percentages and compared with the chi-square test or Fisher’s exact test as appropriate. Participant institutions were classified into four groups (institution Q1 to Q4) using quartiles in descending order of their ECPR cases. The cumulative incidences of clinical outcomes were estimated using the Kaplan-Meier method, and differences among each group were assessed using a log-rank test. The multivariable Cox proportional hazards assumptions were conducted for the primary outcome measure. We selected 11 clinically relevant risk-adjusting variables listed in Table 1, a priori: OHCA causes, IABP use, age, estimated glomerular filtration rate (eGFR), hemoglobin, platelet, C-reactive protein (CRP), lactate, use of antithrombotic agents before admission, catheter laboratory puncture, and participated institution category. These covariates were selected based on clinical relevance and prior literature, not on statistical significance in univariate analysis. We also planned a sensitivity analysis before implementing statistical analyses to confirm the robustness of our findings by adding following covariates to the model; activated partial thromboplastin time (APTT), fibrinogen, albumin, hypertension, diabetes, chronic renal failure, cardiac disease, cerebrovascular disease, bystander CPR. Proportional hazards assumptions for the risk-adjustment variables, including categorized OHCA causes, were evaluated using plots of log (time) versus log (-log(survival)) stratified by the variables and deemed acceptable.  As a sensitivity analysis complementary to Cox models, we estimated restricted mean survival time (RMST) at prespecified horizons (τ = 28, 7, and 3 days) using propensity score overlap weighting targeting the average treatment effect in the overlap population (ATO). Propensity scores were estimated with multinomial logistic regression for OHCA causes (3 levels) and logistic regression for IABP (binary), including the same prespecified covariates as in the Cox models; analyses were conducted on complete cases. For each group, weighted survival curves were obtained with survfit using the overlap weights, and RMST was computed as the area under the weighted event-free survival curve up to τ. Pairwise ΔRMST (difference in RMST) and 95% CIs were obtained from a nonparametric percentile bootstrap (B = 500) with re‑estimation of the propensity scores and weights at each replicate. No weighted log‑rank test was performed for overlap‑weighted curves; inference relied on ΔRMST with bootstrap CIs. For OHCA causes and IABP, the prespecified primary exposure, we applied a fixed‑sequence procedure across τ = 28, 7, and 3 days (two-sided α=0.05 was applied at each step; downstream horizons were formally tested only if the preceding test was significant); otherwise, ΔRMSTs were reported descriptively. Covariate balance after weighting was checked using absolute standardized mean differences (threshold 0.10) and overlap plots.  All P-values were 2-sided, and P < 0.05 was considered significant. Missing values were not imputed and were handled as missing value. All variables used in the multivariate analysis and outcome measures assessed in this analysis were prespecified in the registry. Statistical analyses were performed using JMP software (version 18.0, SAS institute Japan, Tokyo, Japan), and R (version 4.3.3, R Foundation for Statistical Computing, Vienna, Austria). The primary packages included survival, WeightIt, cobalt, and survminer. |  |  |  |  |
| --- | --- | --- | --- | --- | --- | --- | --- | --- |
| Statistical methods  Results | 12 | (*b*) Describe any methods used to examine subgroups and interactions | 9  11-13 | The patients were stratified into three groups based on the underlying causes of OHCA as follows: endogenous cardiac, endogenous non-cardiac, and exogenous.  Statistical analysis  Continuous variables were expressed as median with interquartile range (IQR) or mean and standard deviation and were compared using the Mann-Whitney U test or Student t test based upon their distribution. For comparisons among three groups, the Kruskal-Wallis test was used. Categorical variables were expressed as numbers and percentages and compared with the chi-square test or Fisher’s exact test as appropriate. Participant institutions were classified into four groups (institution Q1 to Q4) using quartiles in descending order of their ECPR cases. The cumulative incidences of clinical outcomes were estimated using the Kaplan-Meier method, and differences among each group were assessed using a log-rank test. The multivariable Cox proportional hazards assumptions were conducted for the primary outcome measure. We selected 11 clinically relevant risk-adjusting variables listed in Table 1, a priori: OHCA causes, IABP use, age, estimated glomerular filtration rate (eGFR), hemoglobin, platelet, C-reactive protein (CRP), lactate, use of antithrombotic agents before admission, catheter laboratory puncture, and participated institution category. These covariates were selected based on clinical relevance and prior literature, not on statistical significance in univariate analysis. We also planned a sensitivity analysis before implementing statistical analyses to confirm the robustness of our findings by adding following covariates to the model; activated partial thromboplastin time (APTT), fibrinogen, albumin, hypertension, diabetes, chronic renal failure, cardiac disease, cerebrovascular disease, bystander CPR. Proportional hazards assumptions for the risk-adjustment variables, including categorized OHCA causes, were evaluated using plots of log (time) versus log (-log(survival)) stratified by the variables and deemed acceptable.  As a sensitivity analysis complementary to Cox models, we estimated restricted mean survival time (RMST) at prespecified horizons (τ = 28, 7, and 3 days) using propensity score overlap weighting targeting the average treatment effect in the overlap population (ATO). Propensity scores were estimated with multinomial logistic regression for OHCA causes (3 levels) and logistic regression for IABP (binary), including the same prespecified covariates as in the Cox models; analyses were conducted on complete cases. For each group, weighted survival curves were obtained with survfit using the overlap weights, and RMST was computed as the area under the weighted event-free survival curve up to τ. Pairwise ΔRMST (difference in RMST) and 95% CIs were obtained from a nonparametric percentile bootstrap (B = 500) with re‑estimation of the propensity scores and weights at each replicate. No weighted log‑rank test was performed for overlap‑weighted curves; inference relied on ΔRMST with bootstrap CIs. For OHCA causes and IABP, the prespecified primary exposure, we applied a fixed‑sequence procedure across τ = 28, 7, and 3 days (two-sided α=0.05 was applied at each step; downstream horizons were formally tested only if the preceding test was significant); otherwise, ΔRMSTs were reported descriptively. Covariate balance after weighting was checked using absolute standardized mean differences (threshold 0.10) and overlap plots.  All P-values were 2-sided, and P < 0.05 was considered significant. |  |  |  |  |
|  |  | (*c*) Explain how missing data were addressed | 13 | Missing values were not imputed and were handled as missing value. |  |  |  |  |
|  |  | (*d*) *Cohort study*—If applicable, explain how loss to follow-up was addressed  *Case-control study*—If applicable, explain how matching of cases and controls was addressed  *Cross-sectional study*—If applicable, describe analytical methods taking account of sampling strategy | - | Not applicable |  |  |  |  |
|  |  | (*e*) Describe any sensitivity analyses | 11-12 | The multivariable Cox proportional hazards assumptions were conducted for the primary outcome measure. We selected 11 clinically relevant risk-adjusting variables listed in Table 1, a priori: OHCA causes, IABP use, age, estimated glomerular filtration rate (eGFR), hemoglobin, platelet, C-reactive protein (CRP), lactate, use of antithrombotic agents before admission, catheter laboratory puncture, and participated institution category. These covariates were selected based on clinical relevance and prior literature, not on statistical significance in univariate analysis. We also planned a sensitivity analysis before implementing statistical analyses to confirm the robustness of our findings by adding following covariates to the model; activated partial thromboplastin time (APTT), fibrinogen, albumin, hypertension, diabetes, chronic renal failure, cardiac disease, cerebrovascular disease, bystander CPR. Proportional hazards assumptions for the risk-adjustment variables, including categorized OHCA causes, were evaluated using plots of log (time) versus log (-log(survival)) stratified by the variables and deemed acceptable.  As a sensitivity analysis complementary to Cox models, we estimated restricted mean survival time (RMST) at prespecified horizons (τ = 28, 7, and 3 days) using propensity score overlap weighting targeting the average treatment effect in the overlap population (ATO). Propensity scores were estimated with multinomial logistic regression for OHCA causes (3 levels) and logistic regression for IABP (binary), including the same prespecified covariates as in the Cox models; analyses were conducted on complete cases. For each group, weighted survival curves were obtained with survfit using the overlap weights, and RMST was computed as the area under the weighted event-free survival curve up to τ. Pairwise ΔRMST (difference in RMST) and 95% CIs were obtained from a nonparametric percentile bootstrap (B = 500) with re‑estimation of the propensity scores and weights at each replicate. No weighted log‑rank test was performed for overlap‑weighted curves; inference relied on ΔRMST with bootstrap CIs. For OHCA causes and IABP, the prespecified primary exposure, we applied a fixed‑sequence procedure across τ = 28, 7, and 3 days (two-sided α=0.05 was applied at each step; downstream horizons were formally tested only if the preceding test was significant); otherwise, ΔRMSTs were reported descriptively. Covariate balance after weighting was checked using absolute standardized mean differences (threshold 0.10) and overlap plots. |  |  |  |  |
|  |  |  |  |  |  |  |  |  |
| Participants | | | | |  | (a) Report numbers of individuals at each stage of study—eg numbers potentially eligible, examined for eligibility, confirmed eligible, included in the study, completing follow-up, and analyzed | 13,  Figure 1 | A participant selection flow chart is shown in Figure 1. Among the 2,157 adult patients with OHCA who received ECPR in SAVE-J II, 1,935 patients were included in this study; 1,417 had endogenous cardiac causes, 305 had endogenous non-cardiac causes, and 213 had exogenous causes. |
| Participants  Descriptive data | 13*  14* | (b) Give reasons for non-participation at each stage | Figure 1 | - |  |  |  |  |
|  |  | (c) Consider use of a flow diagram | Figure 1 | - |  |  |  |  |
|  |  | (a) Give characteristics of study participants (eg demographic, clinical, social) and information on exposures and potential confounders | 13-14  Table 1 | The median follow-up period was 3 (interquartile range [IQR]: 1–18) days, and 36 (IQR: 21–55) days for survivors.  The mean age was 58.9±14.0 years, and 1,597 patients (82.5%) were men (Table 1). Despite being younger than those in the endogenous non-cardiac or exogenous stratum, participants in the endogenous cardiac stratum had more cardiac risk factors and comorbidities, such as hypertension, diabetes mellitus, dyslipidemia, or history of cardiovascular diseases, and had antithrombotic agents prescribed more frequently before admission, and more frequently underwent additional invasive treatment, including coronary angiography, percutaneous catheter intervention (PCI), IABP insertion, and placement of temporary pacemakers. |  |  |  |  |
| Descriptive data  Outcome data | 14*  15* | (b) Indicate number of participants with missing data for each variable of interest | Table 1 | - |  |  |  |  |
|  |  | (c) *Cohort study*—Summarize follow-up time (eg, average and total amount) | 13 | The median follow-up period was 3 (interquartile range [IQR]: 1–18) days, and 36 (IQR: 21–55) days for survivors. |  |  |  |  |
|  |  | *Cohort study*—Report numbers of outcome events or summary measures over time | 14 | Among the current study population, 389 patients (20.1%) developed bleeding complications during hospitalization. Most of the bleeding events occurred within a week after ECPR (median 2 [IQR, 1–8] days). The 30-day cumulative incidence of any bleeding was highest in the patients with endogenous cardiac causes, followed by endogenous non-cardiac and exogenous causes (endogenous cardiac: 321 [25.9%]; endogenous non-cardiac: 41 [18.9%] and exogenous: 27 [13.7%], P<0.001; Figure 2). The incidence of procedure-related bleeding was higher in patients with endogenous cardiac causes (endogenous cardiac: 238 [19.3%]; endogenous non-cardiac: 30 [13.8%] and exogenous: 25 [12.8%], P=0.024) whereas that of non-procedure-related bleeding was higher in patients with endogenous cardiac and non-cardiac causes (endogenous cardiac: 120 [9.8%]; endogenous non-cardiac: 19 [8.7%]; and exogenous: 4 [1.9%], P=0.006). The cumulative 30-day incidence of all-cause death was higher in patients with endogenous non-cardiac causes, followed by those with exogenous and endogenous cardiac causes (endogenous cardiac: 989 [71.0%]; endogenous non-cardiac: 254 [84.8%]; and exogenous: 157 [75.0%], P<0.001). |  |  |  |  |
| Outcome data  Main results | 15*  16 | *Case-control study—*Report numbers in each exposure category, or summary measures of exposure | *-* | Not applicable |  |  |  |  |
|  |  | *Cross-sectional study—*Report numbers of outcome events or summary measures | *-* | Not applicable |  |  |  |  |
|  |  | (*a*) Give unadjusted estimates and, if applicable, confounder-adjusted estimates and their precision (eg, 95% confidence interval). Make clear which confounders were adjusted for and why they were included | 14, 15 | The 30-day cumulative incidence of any bleeding was highest in the patients with endogenous cardiac causes, followed by endogenous non-cardiac and exogenous causes (endogenous cardiac: 321 [25.9%]; endogenous non-cardiac: 41 [18.9%] and exogenous: 27 [13.7%], P<0.001; Figure 2). The incidence of procedure-related bleeding was higher in patients with endogenous cardiac causes (endogenous cardiac: 238 [19.3%]; endogenous non-cardiac: 30 [13.8%] and exogenous: 25 [12.8%], P=0.024) whereas that of non-procedure-related bleeding was higher in patients with endogenous cardiac and non-cardiac causes (endogenous cardiac: 120 [9.8%]; endogenous non-cardiac: 19 [8.7%]; and exogenous: 4 [1.9%], P=0.006). The cumulative 30-day incidence of all-cause death was higher in patients with endogenous non-cardiac causes, followed by those with exogenous and endogenous cardiac causes (endogenous cardiac: 989 [71.0%]; endogenous non-cardiac: 254 [84.8%]; and exogenous: 157 [75.0%], P<0.001).  In a multivariable analysis using the Cox proportional hazard model, the causes of OHCA showed no significant difference in bleeding endpoints after adjustment. However, IABP use was associated with higher risk for any bleeding (hazard ratio [HR] 1.72, 95% confidential interval [CI] 1.27–2.31, P<0.001, Table 2), procedure-related bleeding (HR 1.68, 95% CI 1.21–2.36, P=0.002, Table 2), and non-procedure-related bleeding (HR 2.80, 95% CI 1.60–5.14, P<0.001, Table 2). IABP use was associated with lower risk for all-cause death (HR 0.55, 95% CI 0.47–0.63, P<0.001, Table 2).  Furthermore, IABP use was associated with lower risk for all-cause death (HR 0.55, 95% CI 0.47–0.64, P<0.001, Supplemental Table 1). Age ≥75 years was associated with high risk of any bleeding and procedure-related bleeding, whereas there was no significant difference for non-procedure-related bleeding (Table 2 and Supplemental Table 1). Participating institute category Q4 was associated with a higher risk of any bleeding and non-procedure-related bleeding without no apparent difference for the procedure-related bleeding relative to the category Q1 (Table 2 and Supplemental Table 1). |  |  |  |  |
| Main results | 16 | (*b*) Report category boundaries when continuous variables were categorized | Table 1 | - |  |  |  |  |
|  |  | (*c*) If relevant, consider translating estimates of relative risk into absolute risk for a meaningful time period | - | Not applicable |  |  |  |  |
|  |  |  |  |  |  |  |  |  |

Continued on next page

| Other analyses | 17 | Report other analyses done—eg analyses of subgroups and interactions, and sensitivity analyses | 15, 16 | Sensitivity analysis using 20 covariates confirmed the results; the causes of OHCA causes showed no significant difference in bleeding endpoints after adjustment whereas IABP use was associated with higher risk for any bleeding (HR 1.84, 95% CI 1.33–2.53, P<0.001, Supplemental Table 1), procedure-related bleeding (HR 1.84, 95% CI 1.29–2.66, P=0.001, Supplemental Table 1), and non-procedure-related bleeding (HR 2.87, 95% CI 1.56–5.27, P<0.001, Supplemental Table 1).  After propensity‑score overlap weighting, covariate balance was satisfactory in both the OHCA‑cause and IABP models; nearly all absolute standardized mean differences were <0.10 and none exceeded 0.25 (Supplemental Figure 1). At τ=28 days, overlap‑weighted RMST differences across OHCA causes strata were small for any bleeding and all‑cause death: versus cardiac causes, noncardiac showed ΔRMST −0.25 days (95% CI −3.04 to 2.06) for any bleeding and −0.19 days (−1.97 to 1.42) for death, while exogenous showed +1.91 days (−0.34 to 4.23) for any bleeding and +0.06 days (−1.87 to 2.17) for death (Table 3; Supplemental Figure 2). For procedure‑related bleeding, differences were modest and imprecise (noncardiac −0.08 days, −2.46 to 1.87; exogenous +0.98 days, −1.25 to 3.21). For non‑procedure‑related bleeding, exogenous versus cardiac demonstrated a modest but statistically significant longer bleeding‑free time (ΔRMST +0.96 days, 0.34–1.62), whereas noncardiac versus cardiac was −1.41 days (−3.47 to 0.31). Using IABP as the exposure, RMST indicated shorter bleeding‑free time (ΔRMST [IABP − No IABP] −2.69 days for any bleeding, −2.13 days for procedure‑related, and −1.74 days for non‑procedure‑related; all CIs excluding 0) and longer survival time (ΔRMST +5.58 days, 4.26–6.77) at 28 days (Table 3). Effects at 7 and 3 days were directionally consistent (Supplemental Table 2 and 3). Overlap‑weighted cumulative‑incidence curves were concordant—showing higher bleeding but lower mortality in the IABP group (Supplemental Figure 3). These RMST findings, which do not assume proportional hazards, were concordant in direction with the Cox models and support the robustness of the main results. |
| --- | --- | --- | --- | --- |
| Discussion | | | | |
| Key results | 18 | Summarize key results with reference to study objectives | 17 | To our knowledge, the SAVE-J II registry is the largest study that has evaluated the risks and predictors of hemorrhagic complications in patients who underwent ECPR for OHCA. From this large-scale registry, the main findings of the current analysis were as follows: 1) approximately one-fifth of patients with OHCA experience bleeding complications within a few days after ECPR, and the incidences were different among the causes of OHCA; 2) although the cause of OHCA was not an independent predictor of bleeding, the incidence of bleeding was high in the endogenous cardiac causes group; and 3) additional IABP use on VA-ECMO was an independent risk factor for bleeding, regardless of the types of hemorrhagic complications (Central Illustration). In addition, adjacent IABP use was associated with lower risks for all-cause death. |
| Limitations | 19 | Discuss limitations of the study, taking into account sources of potential bias or imprecision. Discuss both direction and magnitude of any potential bias | 22-24 | Study limitations  Some limitations of the present study should be acknowledged. First, this observational registry data included heterogeneous patient populations without standardized treatment protocols across participating centers. Therefore, we cannot exclude the effect of unmeasured confounding factors that potentially affected our results. Second, due to the retrospective nature of current analysis, we could not use standardized bleeding criteria such as Extracorporeal Life Support Organization (ELSO)34, Bleeding Academic Research Consortium (BARC)35, or GUSTO (Global Use of Strategies to Open Occluded Arteries)36 criteria. However, our bleeding definition corresponds to GUSTO bleeding criteria of moderate or higher bleeding and BARC bleeding criteria of type 3 or higher bleeding. While we might have underestimated minor bleeding, we have evaluated bleeding that requires critical medical intervention in post-ECPR management. Third, the current data set did not include patients with percutaneous ventricular assist devices (PVAD) because they were unavailable during the study period in Japan. Nonetheless, PVAD use has been increasing, and the associated bleeding risk has been reported to be higher than with IABP.37 Therefore, additional evaluation of the bleeding risk of PVAD use in ECPR would be needed. Fourth, although we adjusted for clinically relevant variables, some potentially valuable factors, such as details of anticoagulation management (e.g., activated clotting time targets), timing and volume of blood product administration, and specific technical aspects of ECMO cannulation, were unavailable in our dataset. In addition, we used 11 clinically selected risk adjusting covariates to minimize potential bias, however, there might exist inevitable selection bias. As a sensitivity analysis, we added additional sensitivity analysis using 20 covariates to confirm the results, however, we need other validation analysis to confirm the results. As pre-specified, we primarily used visual inspection of log(-log(survival)) versus log(time) plots, stratified by each variable, however there is a concern of invalid assumption of proportionality of hazards in each covariate. To confirm the result of the current analysis, we performed sensitivity analysis using propensity score and RMST analysis, which is non-parametric, free from proportionality of hazards of covariates. As a result, time‑scale robust RMST analyses at prespecified horizons provided consistent confirmation with the Cox proportional hazards model. Fifth, the definitions of procedure-related or non-procedure-related bleeding were prespecified before the data collection, however, the definition might include classification bias. Finally, the association between IABP use and improved survival should be interpreted cautiously owing to the potential selection bias inherent in the use of registry data. |
| Interpretation | 20 | Give a cautious overall interpretation of results considering objectives, limitations, multiplicity of analyses, results from similar studies, and other relevant evidence | 22-24 | Study limitations  Some limitations of the present study should be acknowledged. First, this observational registry data included heterogeneous patient populations without standardized treatment protocols across participating centers. Therefore, we cannot exclude the effect of unmeasured confounding factors that potentially affected our results. Second, due to the retrospective nature of current analysis, we could not use standardized bleeding criteria such as Extracorporeal Life Support Organization (ELSO)34, Bleeding Academic Research Consortium (BARC)35, or GUSTO (Global Use of Strategies to Open Occluded Arteries)36 criteria. However, our bleeding definition corresponds to GUSTO bleeding criteria of moderate or higher bleeding and BARC bleeding criteria of type 3 or higher bleeding. While we might have underestimated minor bleeding, we have evaluated bleeding that requires critical medical intervention in post-ECPR management. Third, the current data set did not include patients with percutaneous ventricular assist devices (PVAD) because they were unavailable during the study period in Japan. Nonetheless, PVAD use has been increasing, and the associated bleeding risk has been reported to be higher than with IABP.37 Therefore, additional evaluation of the bleeding risk of PVAD use in ECPR would be needed. Fourth, although we adjusted for clinically relevant variables, some potentially valuable factors, such as details of anticoagulation management (e.g., activated clotting time targets), timing and volume of blood product administration, and specific technical aspects of ECMO cannulation, were unavailable in our dataset. In addition, we used 11 clinically selected risk adjusting covariates to minimize potential bias, however, there might exist inevitable selection bias. As a sensitivity analysis, we added additional sensitivity analysis using 20 covariates to confirm the results, however, we need other validation analysis to confirm the results. As pre-specified, we primarily used visual inspection of log(-log(survival)) versus log(time) plots, stratified by each variable, however there is a concern of invalid assumption of proportionality of hazards in each covariate. To confirm the result of the current analysis, we performed sensitivity analysis using propensity score and RMST analysis, which is non-parametric, free from proportionality of hazards of covariates. As a result, time‑scale robust RMST analyses at prespecified horizons provided consistent confirmation with the Cox proportional hazards model. Fifth, the definitions of procedure-related or non-procedure-related bleeding were prespecified before the data collection, however, the definition might include classification bias. Finally, the association between IABP use and improved survival should be interpreted cautiously owing to the potential selection bias inherent in the use of registry data. |
| Generalizability | 21 | Discuss the generalizability (external validity) of the study results | 23 | Third, the current data set did not include patients with percutaneous ventricular assist devices (PVAD) because they were unavailable during the study period in Japan. Nonetheless, PVAD use has been increasing, and the associated bleeding risk has been reported to be higher than with IABP.^37^ Therefore, additional evaluation of the bleeding risk of PVAD use in ECPR would be needed. |
| Other information | |  | | |
| Funding | 22 | Give the source of funding and the role of the funders for the present study and, if applicable, for the original study on which the present article is based | - | Not applicable |

*Give information separately for cases and controls in case-control studies and, if applicable, for exposed and unexposed groups in cohort and cross-sectional studies.

**Note:** An Explanation and Elaboration article discusses each checklist item and gives methodological background and published examples of transparent reporting. The STROBE checklist is best used in conjunction with this article (freely available on the Web sites of PLoS Medicine at http://www.plosmedicine.org/, Annals of Internal Medicine at http://www.annals.org/, and Epidemiology at http://www.epidem.com/). Information on the STROBE Initiative is available at www.strobe-statement.org.
