# Supplementary material for: Predictors of Bleeding Complications After Extracorporeal Cardiopulmonary Resuscitation: Insights From the SAVE-J II Study
Source: JACC Asia. 2025 Dec 19;6(3):314–25. doi: 10.1016/j.jacasi.2025.09.027 (PMC12959310; doi:10.1016/j.jacasi.2025.09.027)
Supplement: Supplemental Figures and Tables [file mmc2.pdf]

Supplemental Materials

Index:

Supplemental Figure Legends: 2
Supplemental Table 1. Sensitivity analysis: Potential risk factors for bleeding and all-cause death 3
Supplemental Table 2. RMST analysis for OHCA causes. 6
Supplemental Table 3. RMST analysis for IABP use 8
Supplemental Figure 1. Propensity-score diagnostics (ATO overlap weighting) 9
Supplemental Figure 2. Cumulative incidence by OHCA cause (overlap weighting) 10
Supplemental Figure 3. Cumulative incidence by IABP use (overlap weighting) .. 11

Supplemental Figure Legends:

Supplemental Figure1. Propensity-score diagnostics (ATO overlap weighting).
(A) Love plot showing absolute standardized mean differences (SMDs) before (unweighted) and after overlap weighting. For the three-level OHCA-cause exposure, points display the maximum absolute SMD across all pairwise treatment comparisons; the vertical reference line at |SMD|=0.10 denotes the conventional threshold for acceptable balance. (B) Propensity-score (PS) overlap distributions before and after weighting, displayed for each treatment category. Improved post-weighting overlap together with SMDs below 0.10 indicate adequate covariate balance for subsequent outcome analyses.
ATO, average treatment effect in the overlap population; IABP, intra-aortic balloon pumping; OHCA, out-of-hospital cardiac arrest; PS, propensity score; SMD, standardized mean difference.
Vertical dashed line at |SMD|=0.10.

Supplemental Figure 2. Cumulative incidence by OHCA cause (overlap weighting).
Weighted curves shown in the sensitivity analyses were presented descriptively; no weighted log-rank test was performed because inference was based on ΔRMST with bootstrap confidence intervals.
Cumulative incidence curves up to 28 days for (i) any bleeding, (ii) procedure-related bleeding, (iii) non-procedure-related bleeding, and (iv) all-cause death, stratified by OHCA cause (cardiac, non-cardiac, exogenous) with overlap weighting applied. Numbers at risk and cumulative event counts are shown beneath each panel at prespecified time points (days 1, 7, 14, 21, and 28).
OHCA, out-of-hospital cardiac arrest.

Supplemental Figure 3. Cumulative incidence by IABP use (overlap weighting).
Weighted curves shown in the sensitivity analyses were presented descriptively; no weighted log-rank test was performed because inference was based on ΔRMST with bootstrap confidence intervals. Cumulative incidence curves up to 28 days for (i) any bleeding, (ii) procedure-related bleeding, (iii) non-procedure-related bleeding, and (iv) all-cause death, stratified by IABP use with overlap weighting applied. Numbers at risk and cumulative event counts are displayed beneath each panel at days 1, 7, 14, 21, and 28.
IABP, intra-aortic balloon pumping.

Supplemental Table 1. Sensitivity analysis: Potential risk factors for bleeding and all-cause death

	Any bleeding		Procedure-related bleeding		Non-procedure-related bleeding		All-cause death	
	HR (95%CI)	P-value	HR (95%CI)	P-value	HR (95%CI)	P-value	HR (95%CI)	P-value
**Endogenous non-cardiac stratum***	0.91 (0.60-1.37)	0.64	0.87 (0.53-1.37)	0.56	1.75 (0.93-3.30)	0.085	1.07 (0.89-1.30)	0.47
**Exogenous stratum***	0.63 (0.36-1.12)	0.95	0.72 (0.38-1.27)	0.27	0.19 (0.03-1.42)	0.11	1.00 (0.79-1.27)	0.98
**IABP use**	1.84 (1.33-2.53)	<0.001	1.84 (1.29-2.66)	0.001	2.87 (1.56-5.27)	<0.001	0.55 (0.47-0.64)	<0.001
**Age ≥75 years**	1.48 (1.05-2.09)	0.033	1.65 (1.11-2.39)	0.014	1.27 (0.69-2.36)	0.44	1.16 (0.96-1.42)	0.13
**eGFR <60 mL/min/1.73m^2**	0.80 (0.61-1.05)	0.11	0.74 (0.55-1.00)	0.053	1.02 (0.63-1.65)	0.93	1.45 (1.24-1.69)	<0.001
**Hemoglobin <11 g/dL**	0.99 (0.74-1.34)	0.97	1.03 (0.73-1.44)	0.84	0.74 (0.43-1.27)	0.28	1.14 (0.97-1.33)	0.11
**Platelat <10×10^4/μL**	1.34 (0.99-1.82)	0.061	1.25 (0.88-1.76)	0.22	1.79 (1.08-2.96)	0.023	1.25 (1.06-1.48)	0.008
**D-dimer ≥5 mg/dL**	1.42 (1.03-1.96)	0.027	1.30 (0.92-1.89)	0.14	1.92 (1.04-3.57)	0.038	1.17 (0.99-1.38)	0.070
**CRP ≥5 mg/dL**	0.84 (0.42-1.66)	0.60	0.86 (0.36-1.75)	0.71	0.77 (0.24-2.53)	0.67	1.06 (0.79-1.42)	0.70
**Lactate**	1.37	0.43	1.40	0.45	0.94	0.94	1.54	0.022

**>5 mmol/L**	(0.60-3.15)		(0.62-4.01)		(0.22-3.99)		(1.04-2.27)	
**Antithrombotic**	0.83	0.37	0.87	0.56	0.83	0.58	0.76	0.012
**agents**	(0.55-1.25)		(0.54-1.37)		(0.43-1.61)		(0.61-0.94)	
**Cath lab puncture**	1.21	0.16	1.12	0.45	1.08	0.75	0.95	0.48
	(0.93-1.58)		(0.83-1.52)		(0.68-1.70)		(0.81-1.10)	
**Institute Q2**†	1.05	0.75	1.00	0.98	1.26	0.36	0.98	0.76
	(0.79-1.39)		(0.72-1.38)		(0.77-2.06)		(0.84-1.14)	
**Institute Q3**†	1.16	0.56	1.06	0.85	1.93	0.074	1.23	0.12
	(0.71-1.88)		(0.57-1.82)		(0.94-3.30)		(0.95-1.60)	
**Institute Q4**†	1.77	0.026	1.39	0.27	2.70	0.006	1.03	0.86
	(1.11-2.83)		(0.74-2.41)		(1.32-5.50)		(0.75-1.41)	
**APTT ≥60 sec**	0.79	0.095	0.85	0.33	0.66	0.10	0.93	0.31
	(0.59-1.04)		(0.62-1.17)		(0.40-1.09)		(0.80-1.07)	
**Fibrinogen**	0.95	0.73	0.91	0.56	1.12	0.66	0.99	0.95
**<200 mg/dL**	(0.72-1.26)		(0.66-1.25)		(0.69-1.81)		(0.85-1.16)	
**Albumin <4 mg/dL**	1.02	0.94	0.98	0.94	1.04	0.91	1.10	0.43
	(0.72-1.26)		(0.61-1.68)		(0.49-2.24)		(0.86-1.41)	
**Hypertension**	0.89	0.42	0.83	0.23	1.29	0.26	1.04	0.58
	(0.68-1.17)		(0.60-1.13)		(0.83-2.01)		(0.90-1.21)	
**Diabetes**	1.12	0.46	1.15	0.44	1.05	0.87	0.98	0.84
	(0.83-1.53)		(0.80-1.61)		(0.62-1.78)		(0.82-1.17)	
**Chronic renal**	0.92	0.79	1.29	0.42	0.31	0.11	0.90	0.53
**failure**	(0.52-1.64)		(0.68-2.25)		(0.07-1.31)		(0.65-1.25)	

**Cardiovascular disease**	1.06 (0.77-1.45)	0.74	0.91 (0.63-1.31)	0.63	1.44 (0.86-3.50)	0.16	1.20 (1.01-1.42)	0.041
**Cerebrovascular disease**	1.34 (0.85-2.11)	0.23	1.33 (0.76-2.18)	0.30	1.74 (0.86-3.50)	0.12	0.93 (0.70-1.23)	0.60
**Bystander CPR**	0.96 (0.76-1.23)	0.77	0.86 (0.66-1.14)	0.30	1.08 (0.71-1.66)	0.71	0.93 (0.81-1.05)	0.25

* Risks relative to the endogenous cardiac causes group.

† Risks relative to the Institute Q1 group.

HR, hazard ratio; CI, Confidence interval; IABP, intra-aortic balloon pumping; eGFR, estimated glomerular filtration rate; CRP, C-reactive protein; APTT, activated partial thromboplastin time; CPR, cardiopulmonary resuscitation.

Supplemental Table 2. RMST analysis for OHCA causes.

Outcome	Comparison group*	τ (days)	ΔRMST (days)	95% CI	RMST cardiac	RMST noncardiac	RMST exogenous
Any bleeding	Noncardiac	28	-0.25	(-3.04 to 2.06)	22.74	22.49	24.66
		7	0.05	(-0.42 to 0.44)	5.92	5.98	6.29
		3	0.03	(-0.10 to 0.14)	2.70	2.73	2.80
	Exogenous	28	1.91	(-0.34 to 4.23)	22.74	22.49	24.66
		7	0.37	(-0.08 to 0.85)	5.92	5.98	6.29
		3	0.10	(-0.02 to 0.22)	2.70	2.73	2.80
Procedure-related bleeding	Noncardiac	28	-0.08	(-2.46 to 1.87)	23.70	23.62	24.68
		7	0.01	(-0.43 to 0.37)	6.12	6.13	6.30
		3	0.02	(-0.09 to 0.12)	2.76	2.78	2.80
	Exogenous	28	0.98	(-1.25 to 3.21)	23.70	23.62	24.68
		7	0.18	(-0.28 to 0.63)	6.12	6.13	6.30
		3	0.04	(-0.08 to 0.16)	2.76	2.78	2.80
Non-procedure-related bleeding	Noncardiac	28	-1.41	(-3.47 to 0.31)	26.70	25.28	27.66
		7	-0.19	(-0.52 to 0.08)	6.75	6.56	6.92
		3	-0.02	(-0.08 to 0.03)	2.93	2.91	2.97
	Exogenous	28	0.96	(0.34 to 1.62)	26.70	25.28	27.66
		7	0.17	(0.04 to 0.31)	6.75	6.56	6.92
		3	0.05	(0.00 to 0.09)	2.93	2.91	2.97
All-cause death	Noncardiac	28	-0.19	(-1.97 to 1.42)	8.05	7.86	8.11
		7	0.04	(-0.37 to 0.43)	3.29	3.33	3.31

	3	-0.03	(-0.17 to 0.12)	2.02	1.99	2.01
Exogenous	28	0.06	(-1.87 to 2.17)	8.05	7.86	8.11
	7	0.02	(-0.45 to 0.53)	3.29	3.33	3.31
	3	0.00	(-0.16 to 0.16)	2.02	1.99	2.01

* reference: cardiac. ΔRMST = RMST(Comparison group) − RMST(cardiac). Positive values indicate longer mean event-free survival in the comparison group. Prespecified fixed-sequence testing across τ=28, 7, and 3 days (two-sided α=0.05); thus, analyses are exploratory and descriptive; no multiplicity adjustment was applied.

Supplemental Table 3. RMST analysis for IABP use.

Outcome	τ (days)	ΔRMST (days)	95% CI	RMST IABP use	RMST No IABP
**Any bleeding**	28	-2.69	(-4.29 to -1.11)	20.89	23.58
	7	-0.61	(-0.92 to -0.32)	5.52	6.13
	3	-0.20	(-0.28 to -0.11)	2.56	2.77
**Procedure-related bleeding**	28	-2.13	(-3.53 to -0.66)	22.40	24.54
	7	-0.45	(-0.74 to -0.16)	5.84	6.29
	3	-0.15	(-0.23 to -0.07)	2.66	2.81
**Non-procedure-related bleeding**	28	-1.74	(-2.71 to -0.82)	25.26	26.99
	7	-0.37	(-0.54 to -0.22)	6.46	6.83
	3	-0.11	(-0.16 to -0.06)	2.84	2.95
**All-cause death**	28	5.58	(4.26 to 6.77)	12.63	7.04
	7	1.75	(1.46 to 2.03)	4.71	2.96
	3	0.67	(0.57 to 0.78)	2.55	1.88

RMST, restricted mean survival time; CI, confidence interval.

Δ RMST = RMST (IABP use group) – RMST (No IABP group). Positive values indicate longer mean event-free survival in the comparison group. Prespecified fixed-sequence testing across τ=28, 7, and 3 days (two-sided α=0.05). Inference is based on ΔRMST with bootstrap 95% CIs; no weighted log-rank test.

Supplemental Figure 1. Propensity-score diagnostics (ATO overlap weighting)

A：Love plot（OHCA, weighted vs unweighted）

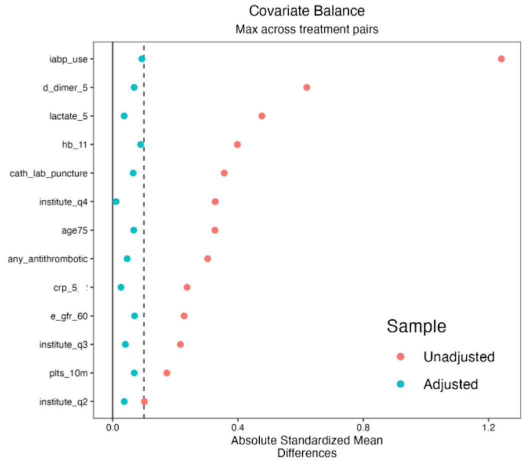

B：PS overlap（OHCA, weighted vs unweighted）

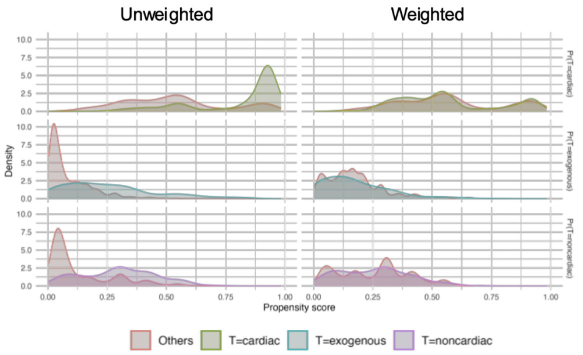

A：Love plot（IABP, weighted vs unweighted）

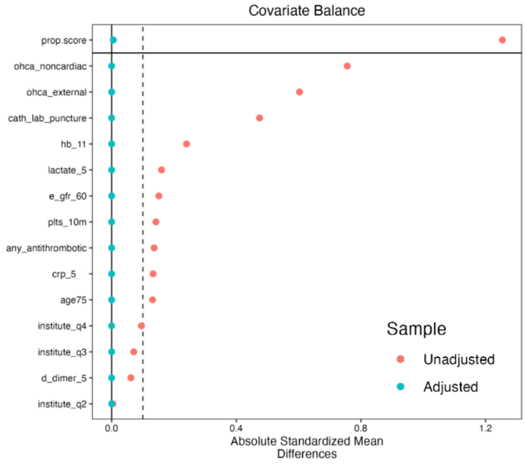

B：PS overlap（IABP, weighted vs unweighted）

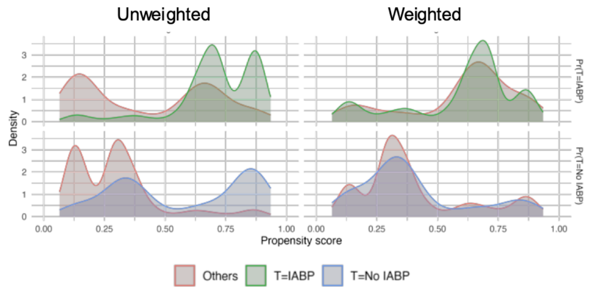

Supplemental Figure 2. Cumulative incidence by OHCA cause (overlap weighting)

Any Bleeding

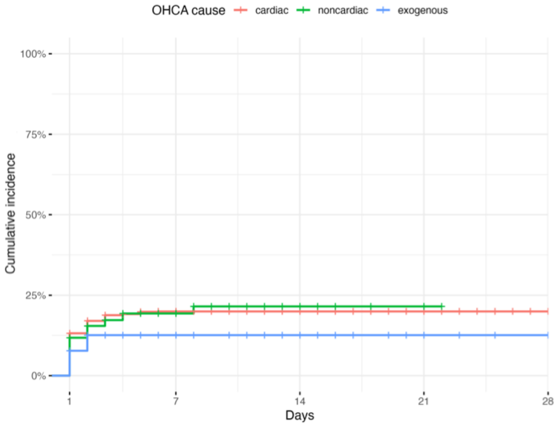

Interval (days)	1	7	14	21	28
Number at risk (cardiac)	1073	304	240	199	165
Cumulative Event (%) (cardiac)	193 (13.1%)	258 (19.9%)	258 (19.9%)	258 (19.9%)	258 (19.9%)
Number at risk (noncardiac)	233	45	30	20	17
Cumulative Event (%) (noncardiac)	27 (11.8%)	34 (19.3%)	35 (21.5%)	35 (21.5%)	35 (21.5%)
Number at risk (exogenous)	157	47	37	26	22
Cumulative Event (%) (exogenous)	14 (7.7%)	17 (12.6%)	17 (12.6%)	17 (12.6%)	17 (12.6%)

Procedure-related bleeding

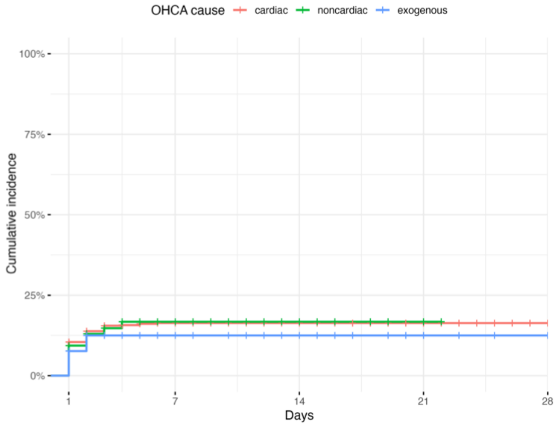

Interval (days)	1	7	14	21	28
Number at risk (cardiac)	1073	330	260	213	172
Cumulative Event (%) (cardiac)	147 (10.4%)	200 (16.3%)	200 (16.3%)	200 (16.3%)	200 (16.3%)
Number at risk (noncardiac)	233	49	33	22	19
Cumulative Event (%) (noncardiac)	19 (9.3%)	26 (16.7%)	26 (16.7%)	26 (16.7%)	26 (16.7%)
Number at risk (exogenous)	157	47	37	26	22
Cumulative Event (%) (exogenous)	13 (7.6%)	16 (12.5%)	16 (12.5%)	16 (12.5%)	16 (12.5%)

Non-procedure-related bleeding

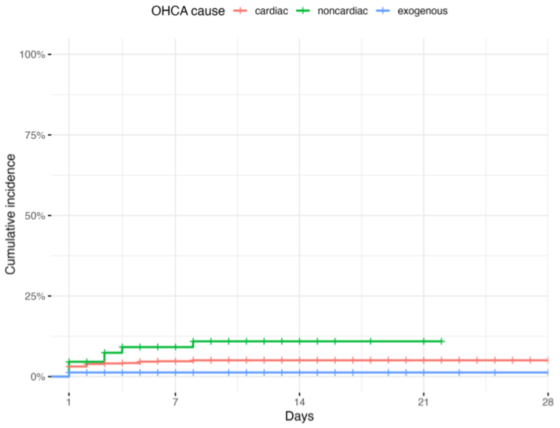

Interval (days)	1	7	14	21	28
Number at risk (cardiac)	1073	406	319	261	215
Cumulative Event (%) (cardiac)	61 (3.1%)	85 (4.8%)	86 (5.0%)	86 (5.0%)	86 (5.0%)
Number at risk (noncardiac)	233	60	43	30	26
Cumulative Event (%) (noncardiac)	13 (4.6%)	15 (9.1%)	16 (10.9%)	16 (10.9%)	16 (10.9%)
Number at risk (exogenous)	157	48	37	26	22
Cumulative Event (%) (exogenous)	2 (1.3%)	2 (1.3%)	2 (1.3%)	2 (1.3%)	2 (1.3%)

All-cause Death

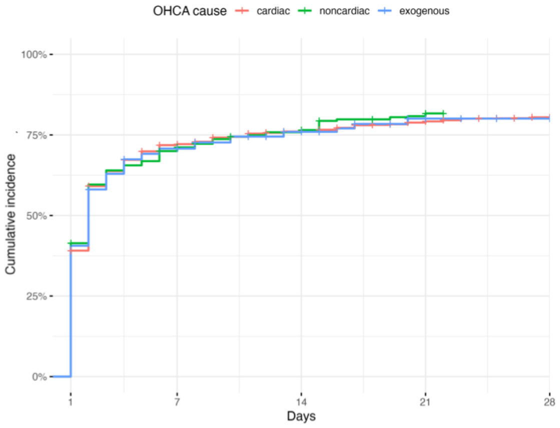

Interval (days)	1	7	14	21	28
Number at risk (cardiac)	1073	446	351	284	230
Cumulative Event (%) (cardiac)	227 (39.1%)	625 (72.1%)	698 (76.5%)	723 (79.2%)	740 (80.5%)
Number at risk (noncardiac)	233	67	48	32	28
Cumulative Event (%) (noncardiac)	101 (41.4%)	167 (71.1%)	180 (76.5%)	190 (81.7%)	190 (81.7%)
Number at risk (exogenous)	157	48	37	26	22
Cumulative Event (%) (exogenous)	65 (40.6%)	106 (70.8%)	111 (76.0%)	115 (80.1%)	115 (80.1%)

Supplemental Figure 3. Cumulative incidence by IABP use (overlap weighting)

Any Bleeding

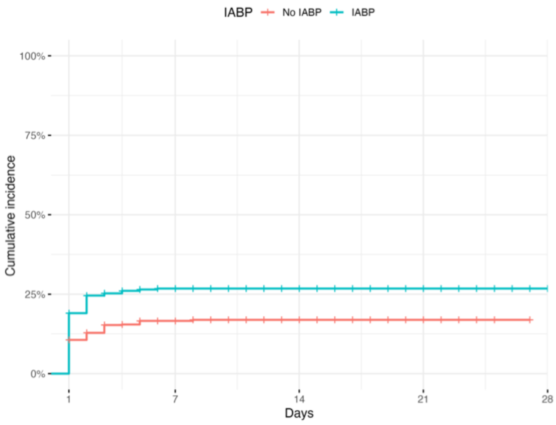

Interval (days)	1	7	14	21	28
Number at risk (No IABP)	592	114	89	66	55
Cumulative Event (%) (No IABP)	60 (10.6%)	73 (16.6%)	74 (16.9%)	74 (16.9%)	74 (16.9%)
Number at risk (IABP)	871	282	218	179	149
Cumulative Event (%) (IABP)	174 (19.0%)	236 (26.8%)	236 (26.8%)	236 (26.8%)	236 (26.8%)

Procedure-related bleeding

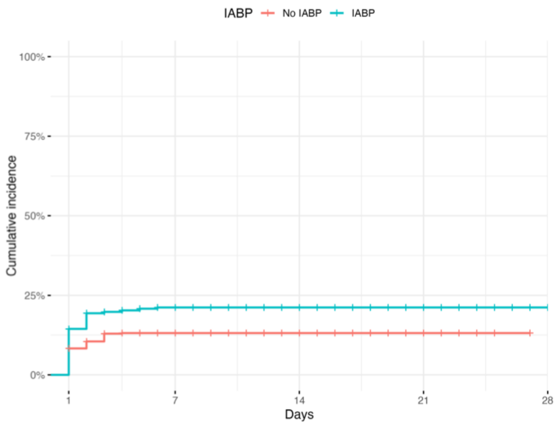

Interval (days)	1	7	14	21	28
Number at risk (No IABP)	592	119	93	68	57
Cumulative Event (%) (No IABP)	48 (8.3%)	60 (13.1%)	60 (13.1%)	60 (13.1%)	60 (13.1%)
Number at risk (IABP)	871	307	237	193	156
Cumulative Event (%) (IABP)	131 (14.4%)	182 (21.1%)	182 (21.1%)	182 (21.1%)	182 (21.1%)

Non-procedure-related bleeding

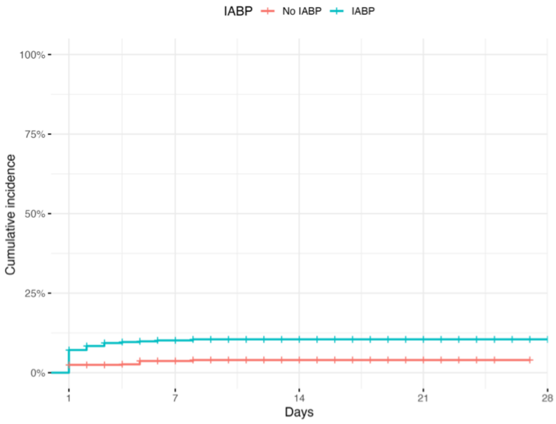

Interval (days)	1	7	14	21	28
Number at risk (No IABP)	592	138	107	80	66
Cumulative Event (%) (No IABP)	15 (2.5%)	17 (3.7%)	18 (4.0%)	18 (4.0%)	18 (4.0%)
Number at risk (IABP)	871	376	292	237	197
Cumulative Event (%) (IABP)	61 (7.1%)	85 (10.1%)	86 (10.5%)	86 (10.5%)	86 (10.5%)

All-cause Death

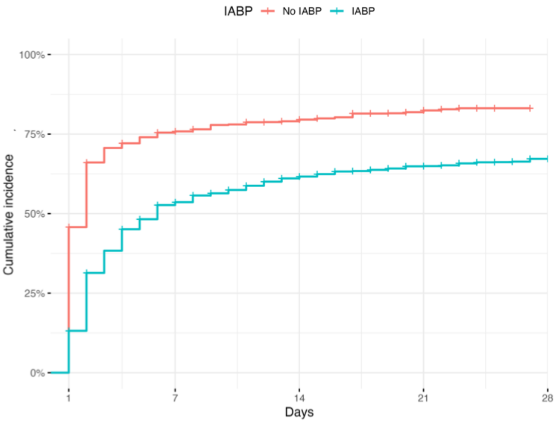

Interval (days)	1	7	14	21	28
Number at risk (No IABP)	592	144	111	82	68
Cumulative Event (%) (No IABP)	281 (45.8%)	443 (75.8%)	465 (79.6%)	481 (82.4%)	483 (83.1%)
Number at risk (IABP)	871	417	325	260	212
Cumulative Event (%) (IABP)	112 (13.2%)	455 (53.6%)	524 (61.7%)	547 (64.9%)	562 (67.2%)
